# Clec16a is Critical for Autolysosome Function and Purkinje Cell Survival

**DOI:** 10.1038/srep23326

**Published:** 2016-03-18

**Authors:** Veronika Redmann, Christopher A. Lamb, Seungmin Hwang, Robert C. Orchard, Sungsu Kim, Minoo Razi, Ashley Milam, Sunmin Park, Christine C. Yokoyama, Amal Kambal, Darren Kreamalmeyer, Marie K. Bosch, Maolei Xiao, Karen Green, Jungsu Kim, Shondra M. Pruett-Miller, David M. Ornitz, Paul M. Allen, Wandy L. Beatty, Robert E. Schmidt, Aaron DiAntonio, Sharon A. Tooze, Herbert W. Virgin

**Affiliations:** 1 Department of Pathology and Immunology, Washington University School of Medicine, St. Louis, MO 63110, USA; 2 Department of Developmental Biology, Washington University School of Medicine, St. Louis, MO 63110, USA; 3 Department of Medicine, Washington University School of Medicine, St. Louis, MO 63110, USA; 4 Genome Engineering and iPSC Center, Washington University School of Medicine, St. Louis, MO 63110, USA; 5 Department of Genetics, Washington University School of Medicine, St. Louis, MO 63110, USA; 6 Department of Molecular Microbiology, Washington University School of Medicine, St. Louis, MO 63110, USA; 7 The Francis Crick Institute, Lincoln’s Inn Fields Laboratory, London, WC2A 3LY, UK; 8 Department of Pathology, University of Chicago, Chicago, IL 60637, USA; 9 Department of Neuroscience, Mayo Clinic Florida, Jacksonville, FL 32224, USA

## Abstract

*CLEC16A* is in a locus genetically linked to autoimmune diseases including multiple sclerosis, but the function of this gene in the nervous system is unknown. Here we show that two mouse strains carrying independent *Clec16a* mutations developed neurodegenerative disease characterized by motor impairments and loss of Purkinje cells. Neurons from *Clec16a*-mutant mice exhibited increased expression of the autophagy substrate p62, accumulation of abnormal intra-axonal membranous structures bearing the autophagy protein LC3, and abnormal Golgi morphology. Multiple aspects of endocytosis, lysosome and Golgi function were normal in Clec16a-deficient murine embryonic fibroblasts and HeLa cells. However, these cells displayed abnormal bulk autophagy despite unimpaired autophagosome formation. Cultured Clec16a-deficient cells exhibited a striking accumulation of LC3 and LAMP-1 positive autolysosomes containing undigested cytoplasmic contents. Therefore Clec16a, an autophagy protein that is critical for autolysosome function and clearance, is required for Purkinje cell survival.

Genome-wide association studies (GWAS) demonstrate that alleles of the *CLEC16A* gene (also KIAA0350) confer risk for autoimmune diseases including type 1 diabetes, multiple sclerosis, primary adrenal insufficiency, primary biliary cirrhosis, alopecia areata, juvenile idiopathic arthritis and rheumatoid arthritis^1^,^2^,^3^,^4^,^5^,^6^,^7^. Studies in *Drosophila* with the homologue of *Clec16a*, *ema*, demonstrate the importance of the gene to the autophagy pathway and protein trafficking in the endosomal pathway^8^,^9^. Autophagy is a cellular degradation process in which cytoplasmic constituents and organelles are enveloped in a double membrane-bound autophagosome which then fuses with the lysosome to create an autolysosome responsible for cargo degradation^10^,^11^,^12^. Autophagy and autophagy proteins play important roles in many biological processes including development, aging, cancer, immunity, inflammation, cell death and cell survival, secretion and neurodegeneration^10^,^13^,^14^,^15^. Clec16a is reported to contribute to the development of diabetes via its role in insulin secretion and, through its interaction with the E3 ubiquitin ligase Nrdp1, to mammalian Parkin-dependent mitophagy^16^. Importantly, *parkin*-deficient mice and mice expressing a dominant negative form of Nrdp1 are not reported to have significant neurodegenerative disease^17^,^18^,^19^. Thus the role of Clec16a in the central nervous system (CNS) has not been defined.

Mice with mutations in the autophagy genes *Atg5*, *Atg7*, *FIP200* and *Epg5* show the importance of autophagy in neurons and their stem cell precursors^20^,^21^,^22^,^23^. Further *in situ* hybridization reveals that cerebellar Purkinje cells and neurons of the deep cerebellar nuclei express high levels of *Clec16a* (Allen Mouse Brain Atlas, http://mouse.brain-map.org/gene/show/50215)^24^,^25^,^26^. Purkinje cells are GABAergic neurons residing between the molecular and granule cell layers of cerebellar folia that help coordinate movement^27^. Purkinje cell axons synapse with neurons in the deep cerebellar nuclei. Interestingly, Purkinje cells lacking the essential autophagy genes *Atg5* or *Atg7* die over time^20^,^21^.

Given the expression of *Clec16a* in the cerebellum and the sensitivity of cerebellar neurons to disruptions in autophagy, we sought to identify the role of *Clec16a* in the CNS of mice. We found that two independent mouse lines carrying homozygous mutations in *Clec16a* exhibited a profound neurodegenerative disease characterized by motor impairment, loss of Purkinje cells, abnormal Golgi morphology, and disrupted autophagy. Abnormalities in Golgi structure and bulk autophagy were also observed in *Clec16a* mutant murine and human cells. Importantly, cultured CLEC16A-deficient cells accumulated autolysosomes despite lysosome and Golgi function being normal by multiple measures and showed normal fusion of autophagosomes and lysosomes. This demonstrated that Clec16a plays a key role in the survival of Purkinje cells in mice and the degradative function or clearance of autolysosomes.

## Results

### Neurologic disease and Purkinje cell loss in *Clec16a* mutant mice

**Figure 1:**
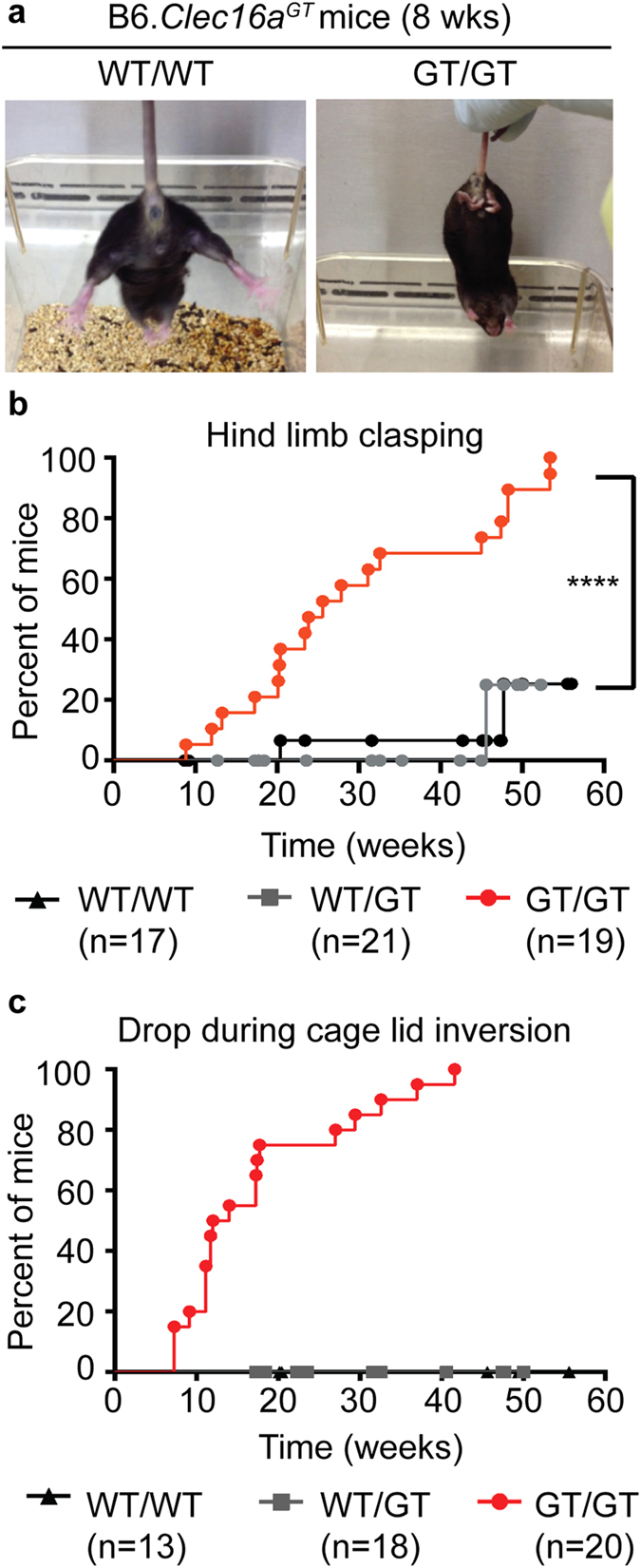
Mutation of Clec16a in mice induces locomotion deficits. (**a**) Image demonstrating normal and aberrant responses hind limb clasping in wild-type and homozygous *Clec16a*^*GT*^ mice. (**b**) Age of mice during the hind limb test when they either scored a 1 (temporary clasping of one or more hind limb to body <30 seconds) or 2 (sustained clasping of both hind limbs to body <30 seconds). (**c**) Age of mice at which they dropped from the cage lid within 20 seconds of the lid being inverted. In (**b**) number of mice/group indicated in each graph; data were analyzed by Log-Rank (Mantel-Cox) test; ****P < 0.0001.

Due to the demonstration in published data of high levels of Clec16a expression seen in Purkinje cells and neurons in the deep cerebellar nuclei (Allen Mouse Brain Atlas, http://mouse.brain-map.org/gene/show/50215)^24^,^25^,^26^, we sought to define the physiological function of *Clec16a* in the central nervous system of mammals by studying mice carrying a gene-trap insertion in *Clec16a (Clec16a*^*GT*^; Fig. 1, Supplementary Fig. 1). *Clec16a*^*GT/GT*^ mice on a mixed 129/SvEv-C57BL/6 genetic background averaged 42% of the weight of control mice (Supplementary Fig. 1b,c) and displayed motor impairment. While many of the Clec16a mutant mice, but not control mice, exhibited hind limb paralysis, we did not separately quantify this observation over time. After backcrossing to the C57BL/6-J background, B6.*Clec16a*^*GT/GT*^ mice continued to display size dimorphism (Supplementary Fig. 1d) and motor impairment. To compare the expression levels of Clec16a transcripts we utilized primers targeting exons 2–3 or exons 23–24 located either 3′ or 5′ of the genetrap cassette, respectively (Supplementary Fig. 1e). While amplification of Clec16a transcripts across exons 2–3 were comparable in wild-type and B6.*Clec16a*^*GT/GT*^ murine embryo fibroblast cells (MEFs), there was a greater than 90% reduction in Clec16a transcript using primers targeting exons 23–24 (Supplementary Fig. 1e), indicating that the mRNA for this gene was interrupted by the GT cassette. The transcription of neighboring genes, *Ciita*, *Dexi* and *Socs-1*, in B6.*Clec16a*^*GT/GT*^ MEFs was unaffected by gene trap insertion in Clec16a (Supplementary Fig. 1e). Starting at seven to eight weeks of age, male and female B6.*Clec16a*^*GT/GT*^ mice displayed abnormal hind limb clasping (Fig. 1a,b)^23^ and were unable to maintain their balance or grip the bars of an inverted metal cage for a normal period of time (Fig. 1c)^28^. To assure that the phenotype observed in B6.*Clec16a*^*GT/GT*^ mice was reflective of disruption of *Clec16a*, we studied a second independent Clec16a mutant mouse strain: *Clec16a*^*CURT*^ mice (on the SWR/J background) carrying a spontaneous 4 base pair deletion in *Clec16a* (Supplementary Fig. 1a)^29^. Transcript levels of Clec16a were significantly reduced in *Clec16a*^*CURT*^ MEFs using primers targeting the mRNA both 5′ and 3′ of the mutation in exon 21, indicating that this mutation significantly destabilizes *Clec16a* mRNA(s) (Supplementary Fig. 1e). These mice also exhibited size dimorphism and motor impairment^29^. Therefore, mutation of *Clec16a* was consistent with the development of neurologic disease in two independent mouse strains.

**Figure 2:**
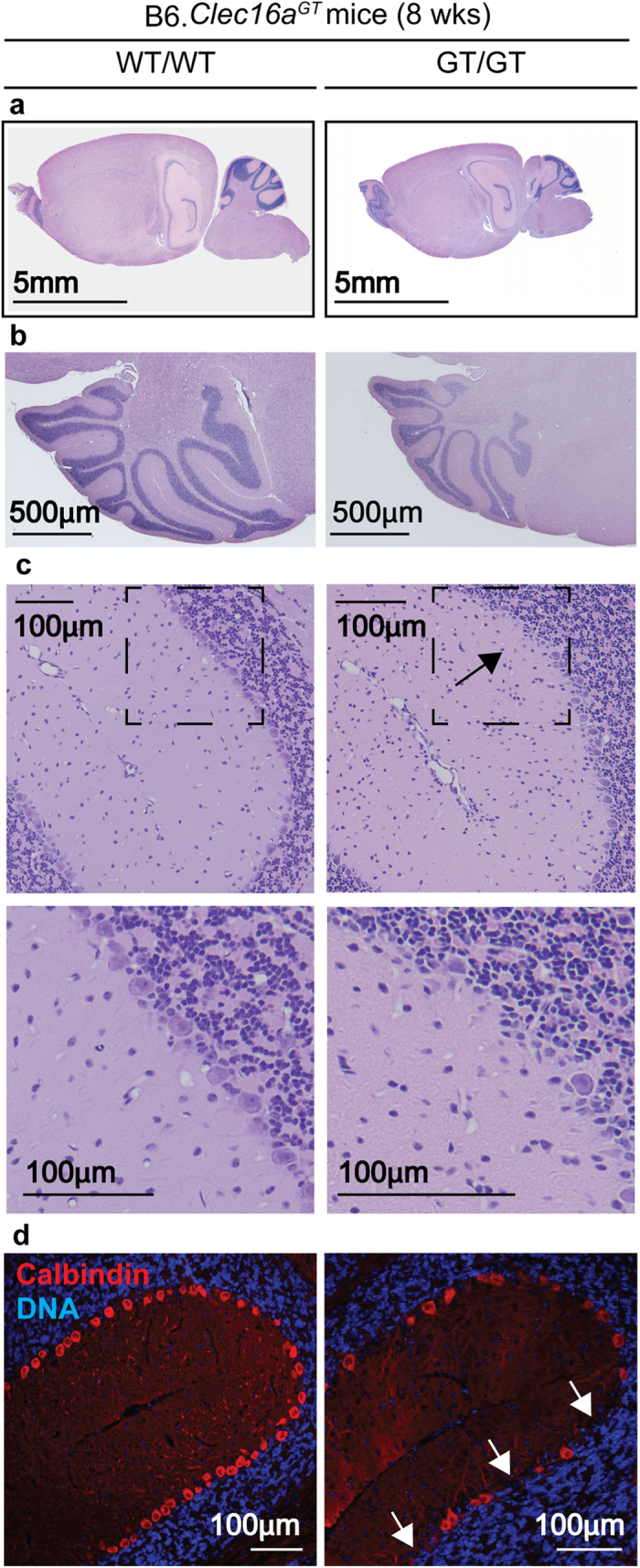
Mutation of Clec16a results in neurodegeneration characterized by loss of Purkinje cells. (**a–d**) H & E (**a–c**) or calbindin immunofluorescence images (**d**) of brains of 8 week old B6.*Clec16a*^*GT*^ mice (representative of n = 3 mice/group). Arrows indicate loss of Purkinje cells.

In order to characterize the specificity of the neurodegeneration seen in B6.*Clec16a*^*GT/GT*^ mice an experienced neuropathologist analyzed multiple areas of the brain, including the pons/medulla, hippocampus, frontal lobe and basal ganglia, and found no evidence that mutation in *Clec16a* resulted in neurodegeneration in these regions of the brain at this time point (Supplementary Fig. 2a–d). However, while gross cerebellar architecture was normal in B6.*Clec16a*^*GT/GT*^ mice despite decreased brain size (Fig. 2a,b), loss of Purkinje cells was readily detectable (Fig. 2c,d) at eight weeks of age before development of neurological deficits in most mice (Fig. 1b,c). Purkinje cell degeneration models such as *pcd* mice display motor abnormalities that were similar to those observed in B6.*Clec16a*^*GT/GT*^ and *Clec16a*^*CURT/CURT*^ mice^30^,^31^ and staining with the Purkinje cell marker calbindin confirmed loss of these cells in B6.*Clec16a*^*GT/GT*^ mice (Fig. 2d) at 8 weeks of age that was dramatically increased by 47 weeks of age (Supplementary Fig. 2e). Bielschowsky staining revealed characteristic ‘empty baskets’ (basket cell processes) in areas of Purkinje cell loss, indicating that the Purkinje cells had developed normally but were then lost (Supplementary Fig. 2f)^32^. *Clec16a*^*CURT/CURT*^ mice also demonstrated loss of Purkinje cells by histology and calbindin staining (Supplementary Fig. 2g–j). These data indicate that *Clec16a* is important for Purkinje cell survival.

### Neuronal and axonal abnormalities in the cerebellum of *Clec16a* mutant mice

**Figure 3:**
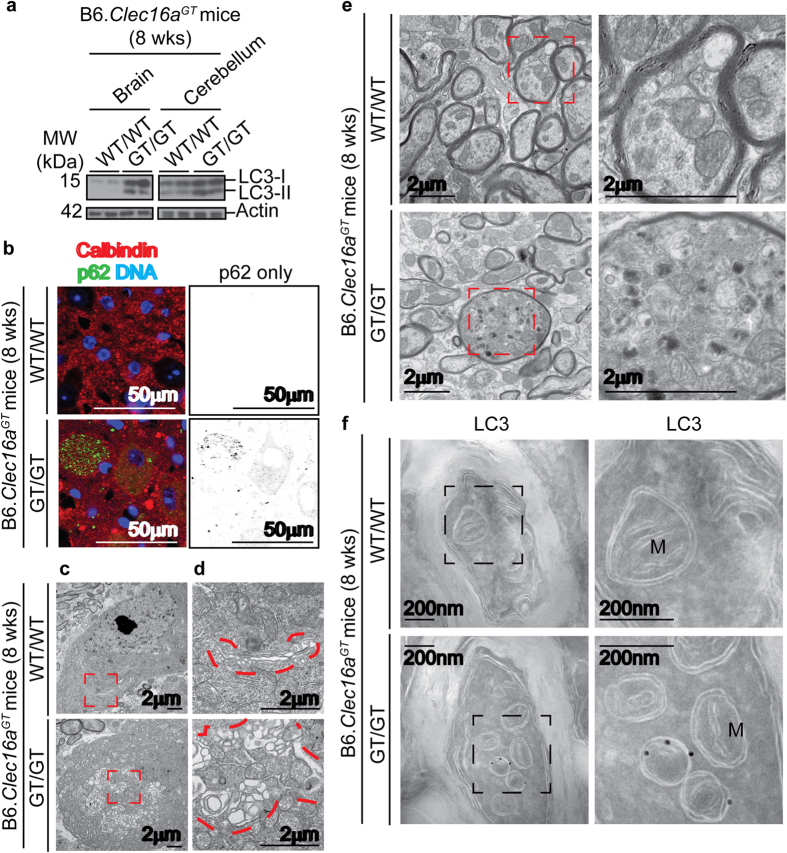
Lack of Clec16a results in LC3 and p62 accumulation and ultrastructural abnormalities in cerebellar neurons. (**a**) Representative western blot of LC3 protein levels in brain or cerebellum from 8 week old B6.*Clec16a*^*GT*^ mice (representative of n = 3 mice/group). (**b–e**) Images of immunofluorescence for calbindin and p62 (**b**) and transmission electron microscopy (**c–e**) from the deep cerebellar nuclei of 8 week old B6.*Clec16a*^*GT*^ mice (representative of n = 3 mice/group). Images highlighting intact Golgi stacks and enlarged endomembrane compartment in neurons (**c**,**d**) and axons (**e**) in the deep cerebellar nuclei. Golgi stacks and compartment outlined in red dotted lines (**d**). (**f**) Images of cryo-immunoelectron microscopy from immunogold labeling for LC3 (12 nm gold) in section of one axon of Purkinje cell of 8 week old B6.*Clec16a*^*GT*^ mice (representative of n = 3 mice/group). Mitochondria are indicated (M).

We selected eight weeks of age, before the onset of severe neurological disease, to examine cerebellar neurons in Clec16a mutant mice in greater detail. Western blots confirmed increased expression of the lipidated form of the autophagy protein LC3 in the cerebellum of B6.*Clec16a*^*GT/GT*^ mice (Fig. 3a). While the cell bodies of Purkinje cells lie in the Purkinje cell layer, axons from Purkinje cells form terminal synapses with neurons in the deep cerebellar nuclei. Neurons in the deep cerebellar nuclei showed accumulation of the autophagy substrate p62 in both B6.*Clec16a*^*GT/GT*^ and *Clec16a*^*CURT/CURT*^ mice (Fig. 3b, Supplementary Fig. 3a). The accumulation of p62 also occurred in calbindin-positive axons, derived from Purkinje cells, in the same region (Fig. 3b, Supplementary Fig. 3a). As electron microscopy is the gold standard for assessment of autophagic structures, we utilized it to assess the effect of Clec16a mutation on the morphology of membrane structures and organelles in Purkinje cells and their axons in the deep cerebellar nuclei. Ultrastructural analysis of mice with *Atg5* and *Atg7* deficiency in Purkinje cells previously demonstrated the accumulation of abnormal membrane structures in the dystrophic axons of Purkinje cells^20^,^21^. We found that cell bodies of cerebellar and deep nuclei neurons from B6.*Clec16a*^*GT/GT*^ mice contained enlarged, vacuolated membranous structures and lacked defined Golgi stacks (Fig. 3c). The morphology of synapses between neuronal processes in both the Purkinje cell layer and the deep cerebellar nuclei were similar between B6.*Clec16a*^*GT/GT*^ mice and littermate controls (data not shown). However in myelinated axons passing through the deep cerebellar nuclei, a region dense in calbindin-positive Purkinje cell axons (Fig. 3b), we observed increased autophagosome- and autolysosome-like structures (Fig. 3e, Supplementary Fig. 3b). These abnormal dystrophic axons were not observed in control mice. The structures within Purkinje cell axons of B6.*Clec16a*^*GT/GT*^ mice were likely of autophagic origin as they labeled for LC3 expression by immuno-electron microscopy (Fig. 3f). Together, these abnormalities suggested a role for Clec16a in both the Golgi apparatus and in autophagy in neurons *in vivo*.

### Accumulation of p62, LC3 in cultured CLEC16A-mutant cells

**Figure 4:**
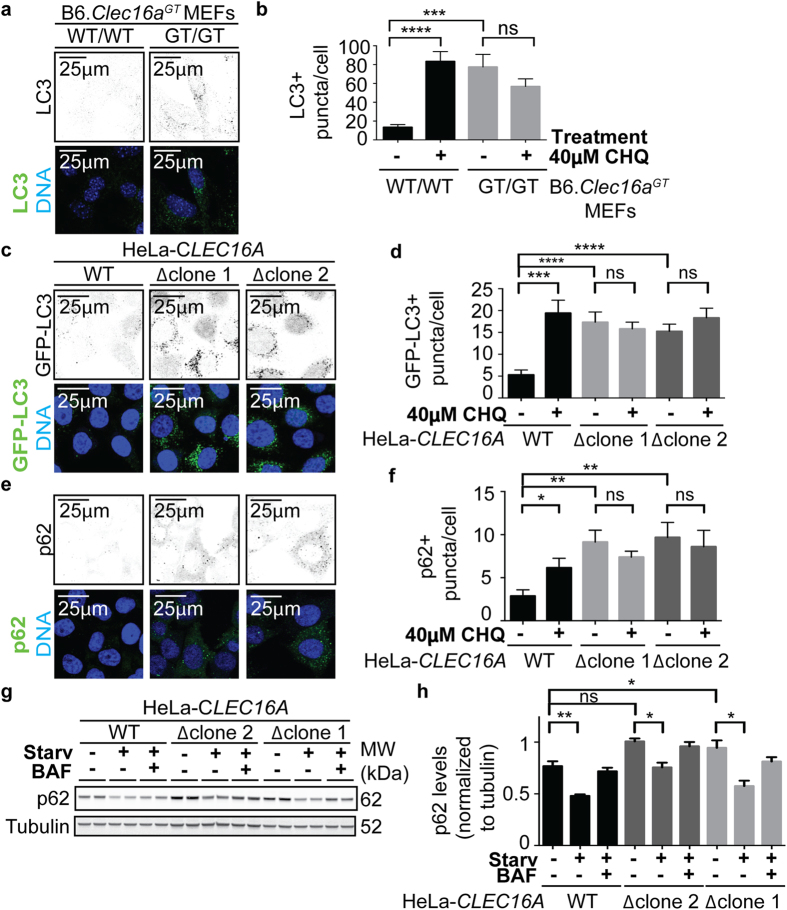
Increased LC3 and p62 in *Clec16a*-mutant MEFs and HeLa cells. (**a**,**b**) B6.*Clec16a*^*GT*^ MEFs, untreated or treated with 40 μm chloroquine were imaged by confocal microscopy (**a**) and the number of LC3+ puncta/cell was quantified (**b**) (representative of n = 3 experiments, minimum 100 cells/condition quantified/experiment). (**c–f**). HeLa-*CLEC16A* cells, untreated or treated with 40 μm chloroquine were imaged by confocal microscopy (**c**,**e**) and the number of GFP-LC3+ (**d**) or p62+ (**f**) puncta/cell was quantified (representative of n = 3–5 experiments, minimum 100 cells/condition quantified/experiment). (**g**,**h**) A representative western blot of p62 in indicated cells starved (Starv) or treated with Bafilomycin A1 (BAF) (**g**), quantified by p62 levels over tubulin (**h**) (representative of n = 3 experiments). Data represent mean+/− s.e.m. and are analyzed by unpaired Student’s t-test; *P < 0.5, **P < 0.01, ***P < 0.001, ****P < 0.0001, ns =  not significant. Scale bars indicate 25μm (**a**,**c**,**e**).

To define the cell biology of the function of Clec16a in autophagy we chose to utilize a rapidly-growing, easily transfectable human HeLa cell line expressing GFP-LC3 to generate CLEC16A-deficient cells using CRISPR/Cas9 nuclease technology. (Supplementary Fig. 4a,b). The use of non-neuronal cell lines, B6.*Clec16a*^*GT/GT*^ MEFs and HeLa-*CLEC16A∆* cells, as oppose to primary cerebellar neuronal cultures, allowed for the extensive quantification and characterization of multiple steps in autophagy by confocal microscopy in cells with abundant cytoplasm. Immunofluorescence analysis of B6.*Clec16a*^*GT/GT*^ MEFs revealed a 5.7 fold increase in LC3+ puncta/cell at baseline compared to control cells and this did not result in a statistically significant change when blocking autolysosome degradation through treatment with chloroquine (CHQ) (Fig. 4a,b). Western blotting demonstrated an increase in p62 and the lipidated form of LC3, termed LC3-II, in B6.*Clec16a*^*GT/GT*^ MEFs (Supplementary Fig. 4c). Similar changes were observed in *Clec16a*^*CURT/CURT*^ MEFs (data not shown). Immunofluorescence analysis of both independent clones of HeLa-*CLEC16A∆* cells demonstrated increased p62+ and LC3+ puncta/cell compared to control cells (Fig. 4c–f) and western blotting revealed increased p62 protein levels in these cells (Fig. 4g,h). To determine whether major changes in cellular energy metabolism might contribute to changes in autophagy, we assessed cellular ATP levels and levels of mitochondrial DNA (mtDNA). We did not detect a change in total mtDNA, or ATP levels in Clec16a-deficient cells, indicating that Clec16a-deficiency was not associated with major alterations in mitophagy in cells cultured under our conditions, and that abnormalities in autophagy were not due to inadequate intracellular energy stores (Supplementary Fig. 4d,e). These data demonstrated impaired bulk autophagy in Clec16a-deficient human and murine cells. The high level of LC3-positive structures at baseline, combined with a failure of chloroquine to increase the number of these structures, was most consistent with Clec16a playing a role in autophagy after the formation of autophagosomes.

### Functional endolysosomal system in *Clec16a*-mutant cells

**Figure 5:**
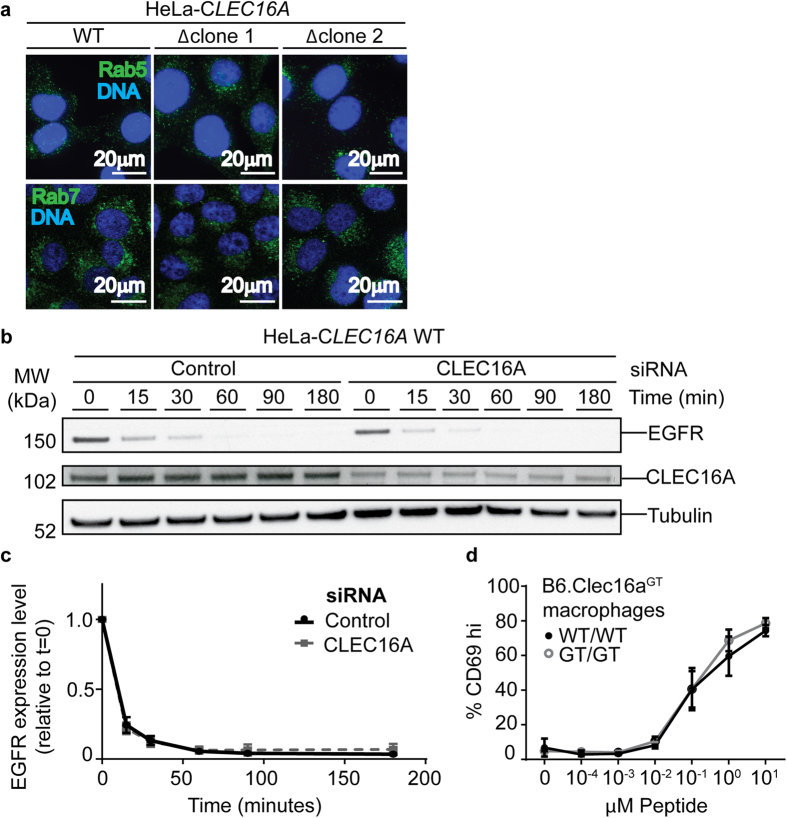
Functional endolysosomal system in Clec16a-mutant cells. (**a**) Confocal microscopy images of HeLa-*CLEC16A* cells stained for Rab5 and Rab7 (representative of n = 3 experiments). (**b**,**c**) EGF receptor degradation assay in control cells or cells with reduced CLEC16A protein by siRNA transfection (representative of n = 3 experiments). (**d**) Co-culture assays measuring T-cell proliferation in response to B6.*Clec16a*^*GT*^ macrophages pulsed with *Listeria monocytogenes*–specific peptide (representative of n = 4 experiments).

Since autophagic flux was abnormal in Clec16a-mutant cells we considered whether another lysosome-dependent degradation process, endolysosomal processing of cell surface proteins, was similarly affected by deletion of Clec16a^9^,^16^,^33^. Knock-down or loss of Clec16a in human and murine cells has been described to alter the morphology of late endosomes^16^,^33^ but we did not observe alterations in the endosomal network as detected by staining of endosomes for Rab5, Rab7 or EEA1 in Clec16a-deficient cells (Fig. 5a, Supplementary Fig. 5a). Knock-down of CLEC16A in HeLa cells reduced the amount of epidermal growth factor (EGF) receptor at steady-state but did not alter the rate of endocytic internalization and endolysosomal degradation of the receptor following ligand binding (Fig. 5b,c). The rate of endolysosomal degradation of the receptor following ligand binding was also unimpaired in CLEC16A-deficient HeLa cells (Supplementary Fig. 5b,c), indicating that a failure to observe changes in siRNA-treated cells was not due to residual low levels of protein. We also examined the cell surface expression of MHC class II which is regulated in part by endosomal recycling of cell surface protein. Presentation of a synthetic peptide antigen to T cells is a sensitive measure of cell surface expression of MHC class II. We therefore assessed the presentation of a peptide derived from *Listeria monocytogenes* by B6.Clec16a^GT*/GT*^ macrophages. Peptide-specific CD4+ T cells co-cultured with peptide-pulsed macrophages from B6.*Clec16a*^*GT/GT*^ mice were equivalently activated across a range of peptide doses indicating normal cell surface expression of functional MHC class II (Fig. 5d). These data are similar to those obtained upon CLEC16A siRNA knockdown in human B cells serving as antigen presenting cells^34^. These assays did not detect abnormalities in the endosomal or endolysosomal system in Clec16a-mutant cells.

### Golgi apparatus structure and function in Clec16a-mutant cells

**Figure 6:**
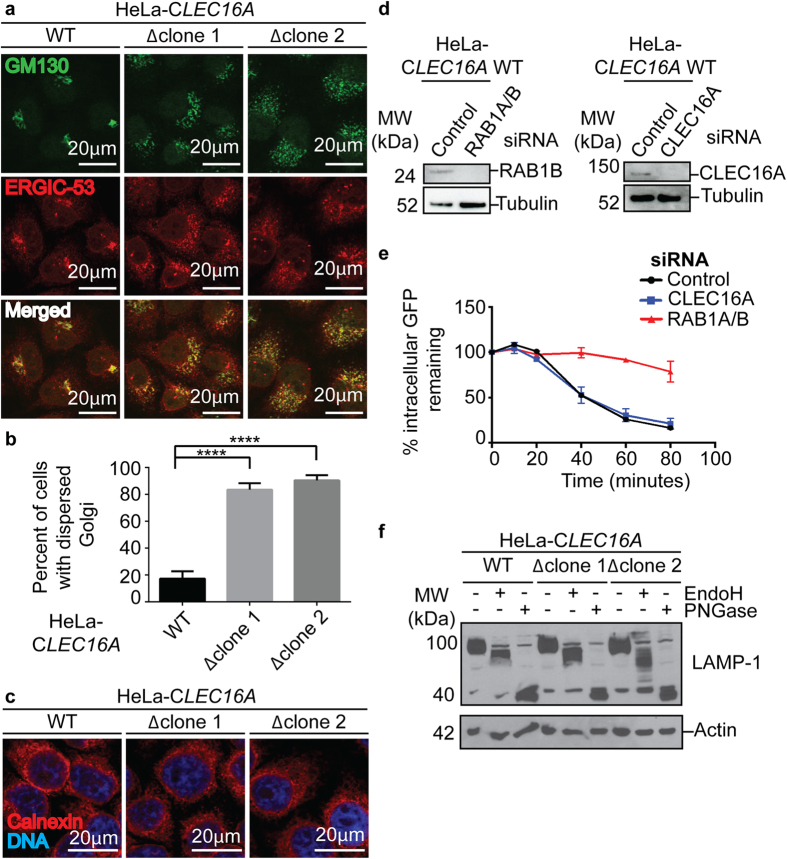
Structure and function of the Golgi apparatus in Clec16a-deficient cells. (**a**–**c**) Confocal microscopy images of HeLa-*CLEC16A* cells stained for GM130, ERGIC-53 (**a**) and calnexin (**c**) and quantification of percent of cells/field with dispersed Golgi apparatus morphology (**b**) (representative of n = 3 experiments, minimum 60 cells/genotype quantified/experiment, data represent mean+/− s.e.m. and were analyzed by unpaired Student’s t-test; ****P < 0.0001). (**d**,**e**) A representative western blot for CLEC16A and Rab1b protein in HeLa C1 transfected cells utilized in hGH-GFP secretion assay (**d**). Remaining intracellular GFP (relative to time = 0) in HeLa C1 cells transfected with control, CLEC16A or RAB1A/B siRNA (E) (representative of n = 2 experiments). (**f**) A representative western blot of LAMP-1 in EndoH and PNGase digested HeLa-*CLEC16A* cells (representative of n = 4 experiments).

Given our novel findings of abnormal Golgi appearance in the neurons of mice lacking *Clec16a*, and the role of the Golgi in production of lysosomes, we next examined the Golgi apparatus in cultured B6.*Clec16a*^*GT*^ and HeLa-*CLEC16A* cells (Fig. 6). CLEC16A-deficient human and mouse cells demonstrated a striking dispersion of the Golgi apparatus and ER-Golgi intermediate compartment (ERGIC) as detected by staining for cis-Golgi matrix protein GM130 and ERGIC protein 53 (ERGIC-53) (Fig. 6a,b, Supplementary Fig. 6a,b). We detected no alteration in endoplasmic reticulum morphology (Fig. 6c), as expected from prior studies of *Clec16a*-deficient murine islets^16^. Staining with other markers such as the trans-Golgi network protein, TGN-46, and the Golgi tethering protein Giantin confirmed the dispersion of the Golgi in CLEC16A-mutant cells (Supplementary Fig. 6c). These abnormalities were not due to defective autophagy in Clec16a-deficient cells since lack of the essential autophagy gene *Atg5* did not lead to altered Golgi or ERGIC morphology (Supplementary Fig. 6d).

Since Clec16a-deficient islet cells are reported to exhibit defective insulin secretion in response to glucose challenge^16^, we quantified protein secretion using HeLa cells expressing a GFP-tagged FKBP mutant (F36M) linked to human growth hormone (hGH)^35^. This protein aggregates in the ER until ligand is added causing solubilization of aggregates and secretion into the extracellular space. In control siRNA-treated cells, intracellular GFP levels declined over time as expected due to hGH secretion (Fig. 6e). Loss of RAB1A/B impaired secretion (Fig. 6d,e), serving as a positive control^36^,^37^. However knock-down of CLEC16A did not affect secretion (Fig. 6d,e).

We next assessed the addition of N-linked glycans to glycoproteins as a measure of Golgi function. Western blotting of EndoH- and PNGase- digested lysates of B6.*Clec16a*^*GT/GT*^ MEFs and HeLa-*CLEC16A∆* cells demonstrated that the highly glycosylated lysosomal membrane protein LAMP-1 was present at equivalent levels in wild-type and CLEC16A-deficient cells. Further, LAMP-1 acquired appropriate N-linked glycans^38^,^39^ (Fig. 6f, Supplementary Fig. 6e). Together these data indicated that the Golgi functions normally in CLEC16A-deficient cells as measured by these assays.

### Clec16a is necessary for autolysosomal function

**Figure 7:**
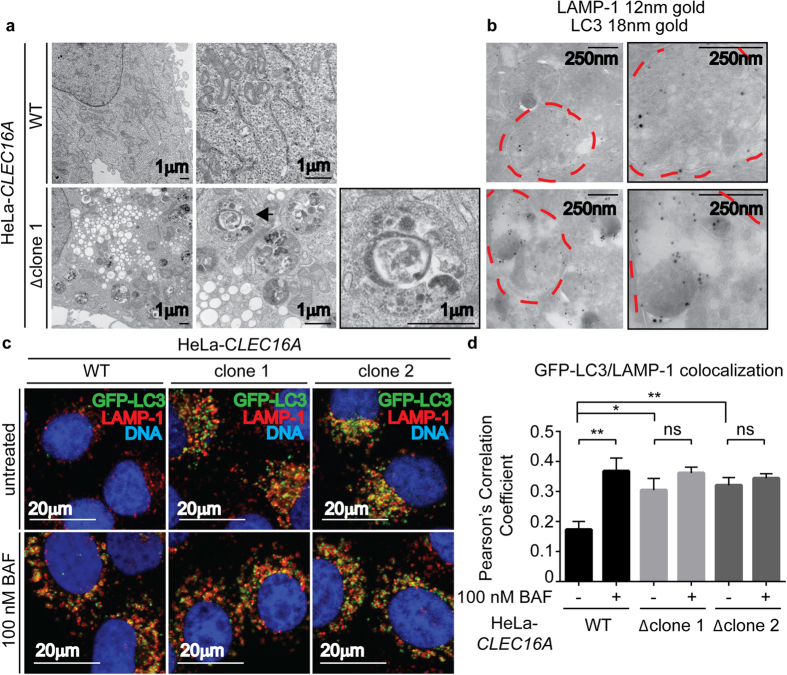
Clec16a-deficient HeLa cells have increased LAMP-1 and LC3 labeled single membrane bound structures. (**a**) Transmission electron microscopy images of HeLa-*CLEC16A* cells (representative of n = 3 experiments). Black arrow indicates membrane bound structures containing cellular debris. (**b**) Cryo-immunoelectron microscopy images of immunogold labeling for LAMP-1 (12 nm gold) and LC3 (18 nm gold) in HeLa-*CLEC16A* cells (representative of n = 3 experiments). Red dotted lines indicate membrane bound structures containing cellular debris. (**c**,**d**) HeLa-*CLEC16A* cells, untreated or treated with 100 nm bafilomycin A1 (BAF), were imaged by confocal microscopy (**c**) and colocalization of GFP-LC3 and LAMP-1 was evaluated by Pearson’s Correlation Coefficient (**d**) (representative of n = 5 experiments, data represent mean+/− s.e.m. and were analyzed by unpaired two-tailed Student’s t-test; *P > 0.5, **P > 0.01; ns =  not significant).

We next investigated the stage in the autophagic process at which Clec16a functions. While in ema-deficient Drosophila cells there is no observed defect in fusion between lysosomes and autophagosomes, reduced fusion has been suggested in studies of mitophagy induction in islet cells lacking Clec16a^9^,^16^. Fusion of LAMP-1+ lysosomes and LC3+ autophagosomes results in the generation of the late autophagic structure, the autolysosome. These structures typically mature into LC3 negative but LAMP-1 positive structures and contain cytoplasmic debris which is ultimately degraded by lysosomal enzymes. B6.*Clec16a*^*GT/GT*^ MEFs and CLEC16A-mutant HeLa cells contained large numbers of single membrane bound structures containing cellular material not seen in control cells (Fig. 7a, Supplementary Fig. 7a,b). These structures were most consistent with being autolysosomes rather than autophagosomes as they had a single membrane. This was confirmed by labeling these structures with both LC3 and LAMP-1 by immuno-electron microscopy (Fig. 7b, Supplementary Fig. 7c).

In order to quantify the increase in autolysosomes seen by electron microscopy we performed colocalization studies with GFP-LC3 and LAMP-1 in CLEC16A-deficient cells. CLEC16A-deficient cells have increased colocalization of LC3 and LAMP-1 in puncta compared to control cells, consistent with the increased number of autolysosomes observed by confocal microscopy (Fig. 7c,d). Importantly, while treatment of control cells with bafilomycin A1 increases the colocalization of LC3 and LAMP-1+ in puncta consistent with its known role in inhibiting lysosome and autophagolysosome function, bafilomycin A1 (BAF) treatment did not increase the colocalization of these markers in CLEC16A-mutant cells (Fig. 7c,d). These data indicate that CLEC16A deficiency results in a failure of autolysosome function or clearance at a step in autophagy downstream of autophagosome and lysosome fusion.

### Lysosomal biogenesis and function in Clec16a-mutant cells

**Figure 8:**
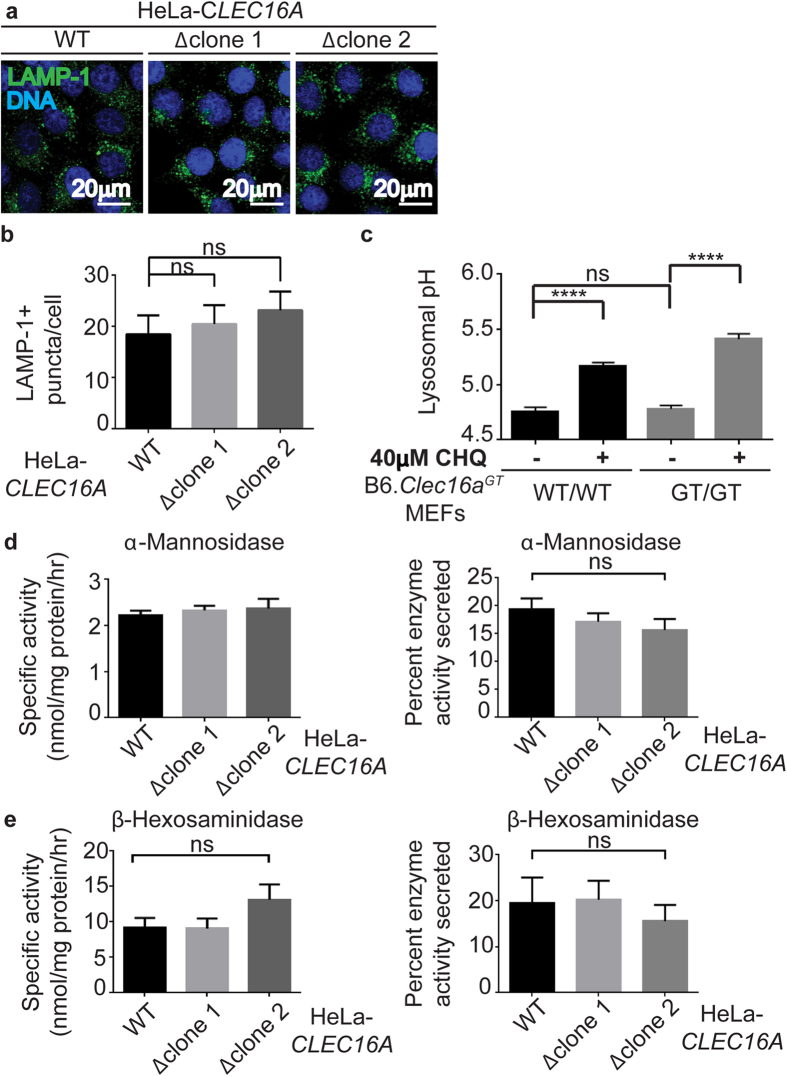
Functional lysosomes in *CLEC16A*-mutant cells. (**a**,**b**) Confocal microscopy images of HeLa-*CLEC16A* cells stained for LAMP-1 (**a**) and quantification of LAMP-1+ puncta/cell (**b**) (representative of n = 5 experiments, minimum 100 cells/genotype quantified/experiment, data represent mean+/− s.e.m. and were analyzed by unpaired Student’s t-test; ns =  not significant). (**c**) Measurement of lysosomal pH with Lysosensor in untreated or chloroquine-treated B6.*Clec16a*^*GT*^ MEFs (representative of n = 3 experiments, data represent mean+/− s.e.m. and were analyzed by unpaired Student’s t-test; ****P > 0.0001, ns =  not significant). (**d**,**e**) Intracellular and extracellular enzyme activity of lysosomal acid hydrolases, α-Mannosidase and β-Hexosaminidase, in HeLa-*CLEC16A* cells (representative of n = 3 experiments, data represent mean +/− s.e.m. and were analyzed by unpaired Student’s t-test, ns = not significant).

Given the failure of autolysosomal clearance, we considered whether Clec16a played a role in lysosomal biogenesis. As described above, we observed normal expression of the lysosomal protein LAMP-1 by western blot analysis in Clec16a-mutant cells (Fig. 6f, Supplementary Fig. 6e). Further, the distribution and number of lysosomes as marked by the lysosomal membrane protein LAMP-1 was not altered by loss of CLEC16A expression in human cells (Fig. 8a,b). We next determined if lysosomal pH is altered in B6.*Clec16a*^*GT*^ MEFs using a pH-sensitive fluorescent sensor. B6.*Clec16a*^*GT/GT*^ MEFs and control cells had a comparable lysosomal pH of 4.8 and as expected this was elevated by treatment with chloroquine (Fig. 8c). Lysosomal acid hydrolases traffic from the Golgi to the lysosome after addition and subsequent enzymatic modification of mannose 6-phosphate (Man-6-P)^40^. Mutations to the first enzyme in this pathway, UDP-GlcNAc: lysosomal enzyme N-acetylglucosamine-1-phosphotransferase, lead to extracellular secretion of hydrolases due to inappropriate glycosylation^41^. However, intracellular and extracellular enzymatic activity of both α-Mannosidase and β-Hexosaminidase was normal in *CLEC16A*-deficient Hela cells, and these enzymes were not secreted (Fig. 8d,e), indicating normal trafficking of these enzymes to lysosomes. These data indicated that defects in autolysosome clearance are not due to major disruptions in lysosomal biogenesis, pH, or hydrolase enzyme trafficking or activity.

## Discussion

We report here that *CLEC16A*, which has been linked by GWAS studies to multiple sclerosis and other autoimmune diseases, has an important role in the survival of cerebellar Purkinje neurons in two mutant mouse models. These results show that one dominant effect of Clec16a deficiency in the whole mouse is related to a role in Purkinje cells. Importantly, the mutations in the gene analyzed here are likely more disruptive of Clec16a function than alleles associated with human disease risk^1^,^2^,^16^,^33^,^42^. Thus these data provide, in a small animal model, insight into the effects of full disruption of the gene in the CNS. Investigation of these cells revealed abnormalities consistent with a role for Clec16a in bulk autophagy, a finding confirmed in both human and murine cells. The block in autophagy occurred after fusion of lysosomes to autophagosomes to create autolysosomes, resulting in accumulation of these late autophagic structures despite normal lysosomal biogenesis and pH. We considered the possibility that abnormalities in autolysosome clearance or function were secondary to abnormal Golgi function or lysosomal biogenesis or function which might correlate with the novel finding of abnormal Golgi morphology detected in our studies, both in neurons and in *in vitro* cell assays. However, protein secretion machinery from the Golgi was unaltered, glycosylation and levels of the lysosomal protein LAMP-1 were normal, the trafficking and activity of lysosomal hydrolases was preserved, and the number and pH of lysosomes was normal in Clec16a-deficient cells. Abnormal Golgi morphology in cells lacking CLEC16A may hint at a possible role of CLEC16A in maintaining or assisting other Golgi apparatus proteins in organelle structure. Together these data argue for abnormal autolysosome accumulation, rather than disrupted Golgi morphology, as the proximate cause of Clec16a-dependent autophagic abnormalities reported here. The specificity of this function of Clec16a for autophagy was further supported by the lack of detectable changes in another lysosomal degradation pathway, the recycling of membrane proteins through the endolysosomal system. We do not know the mechanism by which Clec16a deficiency impairs clearance of autolysosomes. We speculate that the protein is involved in the recycling of autophagic membranes as has been observed to occur late after induction of autophagy^43^. Our findings regarding the loss of Purkinje cells and impaired autophagy point to a potential *in vivo* side-effect of pharmacological inhibition of CLEC16A protein function or autophagy, and support the conclusion that Clec16a has a unique role in the function and clearance of autolysosomes.

### Role of autophagy in Clec16a-dependent neurodegeneration

In several *in vivo* models, neurons, and in particular Purkinje cells, demonstrate a striking requirement for functional autophagy in order to survive. For example, the conditional deletion of the autophagy genes *Atg5* or *Atg7* in Purkinje cells leads to degeneration of these cells^20^,^21^. This phenotype is remarkably similar to what we observed here for mutation of *Clec16a*. Consistent with impairment of autophagy at different steps, axons in the deep cerebellar nuclei of mice lacking *Atg5* and *Atg7* in Purkinje cells displayed increased membranous and vacuole-like structures while Purkinje cell axons in the deep cerebellar nuclei of B6.*Clec16a*^*GT/GT*^ mice accumulated autolysosome-like structures^20^,^21^. Consistent with a protective role of autophagy in neurons, over-expression of Beclin 1 significantly improves motor symptoms in a mouse model of Machado-Joseph disease or spinocerebellar ataxia type 3^44^, and expression of Atg5 protects neurons against virus infection-induced cell death^45^. Roles for several additional autophagy genes in the preservation of other neuronal populations in the CNS include the role of *FIP200* in the neural stem cell population of the lateral ventricles and hippocampal dentate gyrus^22^ and of *Epg5* in cortical layer 5 pyramidal and spinal cord motor neurons^23^. While it is possible that the death of Purkinje cells in mice studied here is due to a non-autophagic role for Clec16a, the similarities between these multiple models of autophagy gene deficiency, together with the role for Clec16a in autolysosomal function and clearance demonstrated here, supports the concept that it is abnormal autophagy, likely as a result of abnormal autophagic flux, that leads to neurodegeneration when Clec16a is mutated. Intriguingly, the Drosophila homologue of Clec16a, ema, has been shown to colocalize with Spinster (*spns1*), which has recently been implicated in abnormal autolysosomal function^46^. Zebrafish with a loss-of-function mutation in *spns1* demonstrate sensitivity to starvation-induced death and accumulate autolysosomes with suboptimal acidification^46^. It is interesting to speculate that Clec16a and other autophagy genes might, in addition to playing a role in survival of specific neurons under normal conditions, participate in survival of neurons in different forms of stress, as for example during auto-immune inflammation in the brain.

### The evolutionarily-conserved role of Clec16a in membrane trafficking

The *Drosophila CLEC16A* homolog *ema* is required for efficient recruitment of the Golgi complex protein Lava lamp (Lva) to autophagic structures in fat body cells and for proper endosomal maturation in Garland cells^9^,^47^. Flies mutant for *ema* exhibit decreased turnover of p62 and decreased mitophagy^9^. Importantly, the phenotype of flies mutant in *ema* can be complemented by expression of human or mouse CLEC16A, indicating that the function of this protein is highly conserved^47^. These studies supported the concept that Ema played a role in autophagosomal growth but that fusion between the autophagosome and the lysosome was unaltered in the absence of the gene^9^. The reasons for the apparent differences between the findings in the fly and our studies are not clear, and will require identification of the molecular mechanisms operating in each system. In contrast, a recent study suggested that impaired mitophagy was due at least in part to reduced fusion between the autophagosomes and lysosomes^16^. It is interesting that this function of Clec16a was attributed to its physical interaction with the E3 ubiquitin ligase Nrdp1 which regulates Parkin. The lack of reported neurodegeneration in *parkin*-deficient and *Nrdp1* mutant mice^17^,^18^,^19^ makes it unlikely that this interaction explains our finding of neurodegeneration in *Clec16a* mutant mice.

### Role of *CLEC16A* in human disease

Studies reported here suggest that a failure to clear autolysosomes and to properly digest their contents plays a role in neuronal survival which may contribute to central nervous system disease. However, single nucleotide polymorphisms (SNPs) in *CLEC16A* that are associated with autoimmunity may correlate with either increased or decreased CLEC16A protein expression in humans, complicating proposals to link the role of Clec16a in autophagy directly to human disease^1^,^2^,^16^,^33^,^42^. For example, the presence of the diabetes-associated *CLEC16A* SNP rs12708716 is reported to result in about a two-fold decrease in CLEC16A mRNA expression in human islets^16^. In contrast, a two- to four-fold increase in CLEC16A mRNA was detected in multiple sclerosis patients compared to controls in peripheral blood mononuclear cells and white matter, though this was not related to the multiple sclerosis risk-associated SNP rs7200786^33^. The importance of these changes in CLEC16A mRNA expression for protein expression and function await analysis. Importantly, the majority of studies on Clec16a function, like our own, have focused on loss of Clec16a function. However, Clec16a plays a fundamentally important role in autophagy by acting at a stage in the process for which specific required genes have not previously been identified. Thus the linkage of this cellular role of Clec16a in disease will be important to delineate.

## Materials and Methods

### Ethics Statement

All animal research was reviewed and approved by the Washington University in St. Louis Institutional Biological and Chemical Safety Committee. All methods were carried out in accordance with the approved guidelines. In order to perform tissue collection, tissues were collected after sacrifice of animals using methods approved by the Animal studies committee approval #20140244.

### Mice and cells

*Clec16a*^*GT*^ mice (Catalog number TF1651) were purchased from Taconic and SWR/J-*Clec16a*^*CURT*^/GrsrJ mice (Stock number 014631, http://mousemutant.jax.org/articles/mmrmutantclec16acurtupdate.html) were purchased from Jackson. *Clec16a*^*GT*^ mice were backcrossed to B6 four generations until microsatellite analysis confirmed complete backcrossing and contain a cre/neo cassette inserted between exon 11 and 12 which initiates Cre expression under the control of the *Clec16a* promoter in place of normal Clec16a expression. Mice were maintained in pathogen-free conditions at Washington University (St. Louis, MO) according to institutional guidelines. HeLa GFP-LC3 cells were a kind gift from B. Levine at UT Southwestern Medical Center^45^. HeLa-CLEC16A^Δclone1^ and HeLa-CLEC16A^Δclone2^ cells were generated by CRISPR nuclease-induced double strand break at the Genome Engineering and iPSC Center at Washington University (St. Louis, MO). HeLa C1 cells were a generous gift from Dr. Andrew Peden, University of Sheffield^35^.

### Behavioural analysis

Tests for hind-limb clasping were performed starting in 4.5 week old B6.*Clec16a*^*GT*^ mice in which a score of 1 was awarded for temporary clasping of 1 or 2 hind limbs and a score of 2 for immediate and sustained clasping of 2 hind limbs over 30 seconds^23^. Data for the hind limb clasping test was then plotted to represent the initial age that individual mice score a 1 or a 2 (whichever occurred first). A drop test was also performed in which mice either dropped immediately when inverted or remained hanging inverted on the cage lid in the first 20 seconds^28^. Drop test data was plotted to represent the age that mice first drop.

### Histology and immunofluorescence studies

B6.*Clec16a*^*GT*^ and *Clec16a*^*CURT*^ mice were perfused with 4% paraformaldehyde and brains were embedded in paraffin prior to H&E staining. For calbindin and p62 brain staining, aged *Clec16a*^*GT*^, B6.*Clec16a*^*GT*^ and *Clec16a*^*CURT*^ mice were perfused with 10% formalin and brains were embedded in paraffin prior to sectioning. Slides were deparaffinized, rehydrated, incubated in ethanol solutions (decreasing from 100–80%) and stained with antibodies. For immunofluorescence studies, B6.*Clec16a*^*GT*^, *Clec16a*^*CURT*^ MEFs and HeLa-*CLEC16A* cells were fixed in 4% paraformaldehyde, permeabilized in methanol, saponin or 0.05% TritonX-100, blocked in 1% BSA and 1% normal goat serum in PBS followed by antibody incubations. Antibodies utilized are listed in Supplementary Information. For quantification of LC3+, LAMP-1+ or p62+puncta/cell and colocalization studies, collective images of at least 100 cells/sample were analyzed using ImageJ or Volocity software. For Golgi organelle morphology scoring, collective images of at least 60 cells/sample were analyzed visually to obtain the percent of cells compared total cells/field with an abnormal Golgi morphology.

### Electron microscopy and immunogold labeling

For transmission electron microscopy, B6.*Clec16a*^*GT*^ and *Clec16a*^*CURT*^ mice were transcardially infused with saline followed by 4% paraformaldehyde and brain tissue sections were fixed and prepared for grids. A minimum of 100 Purkinje cells or neurons of the deep cerebellar nuclei/grid were evaluated. A primary evaluation of ultrastructural morphology was performed on Purkinje cell bodies, axons and cell bodies of neurons of the deep cerebellar nuclei to identify any abnormal pathology. Additionally, the morphology of synapses between neuronal processes were evaluated in both the Purkinje cell layer and deep nuclei to assess any reductions in the number of synapses in the tissues or ultrastructual differences in the synapse. For unbiased evaluation of intracellular membranes in cerebellar neurons, 2 ultrastructural experts were provided with 30 unlabeled electron microscopy images of Purkinje cell bodies and cell bodies of neurons in the deep cerebellar nuclei at a resolution which the morphology of mitochondria, ER, Golgi and vesicles could be evaluated. For transmission electron microscopy of tissue culture cells, cells were fixed, postfixed, dehydrated, embedded and mounted as above for murine tissue. For immunogold labeling of LC3 in murine tissues, B6.*Clec16a*^*GT*^ and *Clec16a*^*CURT*^ mice were pericardially infused with saline followed by 4% paraformaldehyde/0.05% glutaraldhyde (Polysciences Inc., Warrington, PA) in 100 mM PIPES buffer and brain tissue sections were embedded in gelatin, infiltrated with PVP-sucrose overnight and frozen in liquid nitrogen. Sections labeled for LC3 were visualized with transmission electron microscopy. For immunogold labeling of LC3 and LAMP-1, HeLa-*CLEC16A* cells were prepared as above for murine tissues. Sections were labeled for GFP and LAMP-1.

### Secretion, glycosylation assays

HeLa C1 cells^35^ were transfected on days 1 and 2 post-plating with 50 nM of RISC Free control siRNA (D-001220-01), CLEC16a siRNA (D-022485-18) or a pool of siRNA directed against RAB1A and B (L-008283-00, L-008958-01) (Dharmacon/GE Healthcare) using Oligofectamine (day 1) (Life technologies) and Lipofectamine 2000 (day 2) (Life technologies) and re-plated into 6-well cluster plates (day 3). Following treatment with 1 μM D/D solubiliser (FKBP AP21998) (Clontech), the amount of the eGFP tagged reporter construct remaining at each time point was measured by flow cytometry on a LSR II (Beckton Dickinson) and expressed as a percentage of the zero time point for each. For analysis of LAMP-1 glycosylation levels, B6.*Clec16a*^*GT/GT*^ MEFs and HeLa-*CLEC16A* cells were lysed in 1% SDS, 10× Denaturing buffer (New England Biolabs) and lysates were incubated with Endoglycosidase H and Peptide-N Glycanase at 37 °C for 5 hours. Lysates were probed for LAMP-1 protein levels following resolution by SDS-PAGE.

### Lysosomal and mitochondrial function assays

For EGFR degradation assays, protein levels of EGFR were determined by western blot in HeLa cells untransfected, transfected with siRNAs, serum starved and/or stimulated with 100 ng/ml EGF (Sigma-Aldrich, St. Louis, MO) for the indicated times. For measurement of lysosomal pH, B6.*Clec16a*^*GT*^ MEFs were incubated with 4 uM Lysosensor Yellow/Blue DND-160 (Life Technologies, Cat No. L-7545) and, following measurement by plate reader, lysosomal pH was determined with the following ratio of the fluorescence at two different wavelengths: (F_340nm_/F_380nm_). Enzymes activity assays, for β-hexosaminidase, α-mannosidase and β-glucuronidase, were performed as described^48^. For antigen presentation co-culture assays wild-type (BL6) and Clec16a-deficient bone-marrow derived macrophages were peptide pulsed (Listeriolysin O (190–205), 0–10 μM). Splenic CD4 T cells from LLO56tg mice, whose TCR is specific for LLO_190–205_^49^, were isolated (CD4+ T Cell Isolation Kit II – mouse, Miltenyi Biotech) and added to the macrophages at a density of 2.5 × 10^5^ T cells/well. After 24 hours, CD4 cells (eFluor450, Clone RM4–5, eBioscience) were assayed for activation by staining for expression of CD69 (PECy7, Clone H1.2F3, BioLegend). For measurement of cellular ATP levels, B6.*Clec16a*^*GT*^ MEFs and HeLa-*CLEC16A* cells were seeded in 96 well plates. Following addition of CellTiter-Glo Luminescent Cell Viability Reagent (Promega Corporation, Madison, WI) to the plate luminescence was measured using a Molecular Devices SpectraMax M2 plate reader. For mtDNA quantification, qPCR was performed using total DNA from B6.*Clec16a*^*GT*^ MEFs as described^50^. Results were comparable with 100 ng, 10 ng and 1 ng of input DNA.

### Statistical tests

Statistical tests, one way ANOVA, Log-Rank (Mantel-Cox) test or unpaired two-tailed Student’s t-test, were performed using Graphpad Prism software. All bar graphs represent mean +/− s.e.m.

Detailed materials and methods can be found in Supplementary Information.

## Additional Information

**How to cite this article**: Redmann, V. *et al.* Clec16a is Critical for Autolysosome Function and Purkinje Cell Survival. *Sci. Rep.* **6**, 23326; doi: 10.1038/srep23326 (2016).

## Supplementary Material

Supplementary Information
