# Supplementary material for: Clec16a is Critical for Autolysosome Function and Purkinje Cell Survival
Source: Sci Rep. 2016 Mar 18;6:23326. doi: 10.1038/srep23326 (PMC4796910; doi:10.1038/srep23326)

Clec16a is Critical for Autolysosome Function and Purkinje Cell Survival

Veronika Redmann, Christopher A. Lamb, Seungmin Hwang, Robert Orchard, Sungsu Kim, Minoo Razi, Ashley Milam, Sunmin Park, Christine C. Yokoyama, Amal Kambal, Darren Kreamalmeyer, Marie K. Bosch, Maolei Xiao, Karen Green, Jungsu Kim, Shondra M. Pruett-Miller, David Ornitz, Paul M. Allen, Wandy L. Beatty, Robert E. Schmidt, Aaron DiAntonio, Sharon A. Tooze, Herbert W. Virgin

Supplementary information

Supporting Materials and Methods

Cells, antibodies and reagents

HeLa-CLEC16A$^{\Delta clone1}$ and HeLa-CLEC16A$^{\Delta clone2}$ cells were generated by targeting exon 1 of *CLEC16A* with a guide RNA, 5' ggccgctcgcggagctgggtggg 3' (Supplemental Experimental Procedures, Supplemental Figure 3A) and selecting for clones in which aberrant non-homologous end joining resulted in insertions and deletions at the CRISPR nuclease-induced double strand break. These “indels” produced premature stop codons in exon 2 of both gene alleles. Sequencing was used to confirm the absence of two wild-type alleles and the disruption of the open reading frame in both copies of the mutant allele. Parental cell line was used as control for all experiments.

The following antibodies used in this study were: goat anti-CLEC16A (MyBiosource, Inc, San Diego, CA), rabbit anti-LC3b (Cell Signaling Technology, Inc., Danvers, MA; Sigma-Aldrich, St. Louis, MO), mouse anti-calbindin (Sigma-Aldrich, St. Louis, MO), guinea pig anti-p62 (Progen Biotechnik, Cologne, Germany), goat anti- GFP (Abcam,

Cambridge, MA), rabbit anti-SOD1 (Abcam, Cambridge, MA), mouse anti-tubulin (Life Technologies, Grand Island, NY), mouse anti-Actin (Sigma-Aldrich, St. Louis, MO), rabbit anti-Giantin (Covance, Princeton, NJ), mouse anti-GM130 (BD Biosciences, San Jose, CA), rabbit anti-ERGIC-53/p58 (Sigma-Aldrich, St. Louis, MO), rabbit anti-TGN-46 (AbD Serotec), rabbit anti-EEA1 (Cell Signaling Technology, Inc., Danvers, MA), rabbit anti-Rab5 (Cell Signaling Technology, Inc., Danvers, MA), rabbit anti-Rab7 (Cell Signaling Technology, Inc., Danvers, MA), rabbit anti-LAMP-1 (Abcam, Cambridge, MA), rabbit anti-Rab1b (Santa Cruz, Heidelberg, Germany), rabbit anti-TOM20 (Santa Cruz, Heidelberg, Germany) and rabbit anti-EGFR (Cell Signaling Technology, Danvers, MA). AF8 ascites (anti-human calnexin) were a kind gift of M. Brenner (Brigham & Women's Hospital, Boston, MA), Additional microscopy reagents included ToPro (Life Technologies, Grand Island, NY) and ProLong Gold Antifade reagent (Life Technologies, Grand Island, NY).

qRT-PCR assays for RNA transcript copies

Briefly, RNA was extracted by Trizol preparation from B6.*Clec16a*^{GT} and *Clec16a*^{CURT} MEFs. cDNA was prepared using ImProm II (Promega) and Real time qPCR was performed using Taqman or SYBR Green. Taqman assays were ordered from IDT for *Ciita*, (Mm.PT.58.41742531), *Dexi* (Mm.PT.58.41557218), *Clec16a* targeting exons 23-24 (Mm.PT.58.28788735), and *Socs-1* (Mm.PT.58.11527306.g). RNA copies were quantified by standard curves using target sequencing containing gBlocks (IDT) and normalized to *β-actin* (forward primer: 5'-GCTCCTTCGTTGCCGGTCCA-3', reverse primer: 5'-TTGCACATGCCGGAGCCGTT-3', probe: 5'-6-JOEN-

CACCAGTTC/ZEN/GCCATGGATGACGA-IABkFQ-3’). SYBR green qRT-PCR was performed for *Clec16a*, targeting exons 2-3 with the following primers: (5’-GGGGCGATCAAAATGACAGC-3’ and 5’-CTGCACACACACATAACGGC-3’)

Autophagy assays and Immunofluorescence

Briefly, B6.*Clec16a*^{GT} and *Clec16a*^{CURT} MEFs and HeLa-*CLEC16A* cells were harvested in mammalian lysis buffer, vortexed and spun down. Cell lysate was diluted in 2x Laemmli buffer and boiled before separation on a 10% SDS-PAGE gel followed by a wet transfer. For p62, LC3 dot quantification, B6.*Clec16a*^{GT}, *Clec16a*^{CURT} MEFs and HeLa-*CLEC16A* cells were fixed in 4% paraformaldehyde, permeabilized in methanol and blocked in 1mM EDTA, 0.2% TritonX-100, 1% BSA, 1% normal goat serum in PBS. Cells were then incubated with anti-p62 or anti-LC3, then anti-rabbit FITC. B6.*Clec16a*^{GT}, *Clec16a*^{CURT} MEFs and HeLa-*CLEC16A* cells were stained for nuclei and mounted. Images were collected using a ZEISS LSM 510 META confocal laser scanning microscope and LSM 510 Examiner software. The number of p62+, LC3+ puncta/cell were quantified using Image J software. Following comparisons of manual and automatic threshold parameters during assay optimization, Image J autothreshold setting, maxentropy was used for labeling and quantifying number of puncta/image. Cell counter plugin was used to count ToPro marked nuclei. All data was analyzed using Prism software (Graphpad, San Diego, CA) using an unpaired student's t- test. Error bars represent standard error of the mean. Colocalization analysis, via determination of Pearson's Correlation Coefficient/image or quantification of single or double-positive puncta/cell, was performed on confocal images using Volocity software (Perkin-Elmer, Waltham, MA). Scoring of immunofluorescence images for dispersed Golgi apparatus

morphology was performed by counting percent of cells with dispersed morphology/field.

For calbindin staining of brain sections from aged *Clec16a*^{GT}, slides were incubated with ReVeal heat retrievel buffer (Biocare Medical) in a steamer following ethanol washes and then incubated with antibodies and hematoxylin. Slides are washed subsequently in ethanol solutions (increasing from 80-100%) and xylene then mounted.

Electron microscopy and immunogold labeling

For transmission electron microscopy, B6.*Clec16a*^{GT} and *Clec16a*^{CURT} mice were transcardially infused with saline followed by 4% paraformaldehyde, brains were incubated for several hours in modified Karnovsky's fixative containing 3% glutaraldehyde and 1% paraformaldehyde in 0.1M sodium cacodylate buffer pH 7.4. Brains were dissected and tissue sections from the cerebellar DCN and folia were rinsed in 0.1M sodium cacodylate buffer. These sections were postfixed in sodium cacodylate-buffered 2% OsO_4 for 1 hour and dehydrated in graded ethanols with a final dehydration step in propylene oxide. Tissues were embedded in Embed-812 (Electron Microscopy Sciences, Hatfield, PA) and one micron thick plastic sections were stained with toluidine blue and examined by light microscopy before 90 nm thin sections were cut onto formvar coated slot grids. Sections were post-stained with uranyl acetate and Venable's lead citrate and images were collected with a JEOL model 1200 EX electron microscope (JEOL, Tokyo, Japan). AMT Advantage HR (Advanced Microscopy Techniques, Danvers, MA) was used for acquisition of digital images.

For transmission electron microscopy of tissue culture cells, cells were seeded on Permanox slides (Electron Microscopy Sciences, Hatfield, PA) and following desired growth time were incubated for several hours in modified Karnovsky's fixative containing 3% glutaraldehyde and 1% paraformaldehyde in 0.1M sodium cacodylate buffer pH 7.4. For immunogold labeling of LC3 in tissues, B6.*Clec16a*^{GT} and *Clec16a*^{CURT} mice were pericardially infused with saline followed by 4% paraformaldehyde/0.05% glutaraldhyde (Polysciences Inc., Warrington, PA) in 100mM PIPES buffer. Tissues were then embedded in 10% gelatin and infiltrated overnight with 2.3M sucrose/20% polyvinyl pyrrolidone in PIPES at 4 degrees C. Tissues were then frozen in liquid nitrogen and a cryo-ultramicrotome was used for sectioning. Sections were incubated with anti-LC3b (Sigma-Aldrich, St. Louis, MO) and then secondary anti-rabbit conjugated to 18nm colloidal gold. Sections were stained with uranyl acetate/methylcellulose and visualized with transmission electron microscopy. For immunogold labeling of LC3 and LAMP-1, HeLa-*CLEC16A* were fixed in 4% paraformaldehyde/0.05% glutaraldhyde (Polysciences Inc., Warrington, PA) in 100mM PIPES buffer and prepared as above for murine tissues. Sections were incubated with Goat anti-GFP (Abcam), secondary donkey anti-goat (18nm gold) and Rabbit anti-LAMP-1 (kind gift of the Sibley lab, Washington University in St. Louis), secondary donkey anti-rabbit (12nm gold).

Secretion assay in HeLa C1 cells

HeLa C1 cells (Gordon et al 2010) were plated at a density of 1 x10^6 cells per ten cm dish. Following siRNA transfection on days 1 and 2, the cells were then plated into 6-well cluster plates at a density of 4 x 10^5 cells per well. The cells were treated at the

indicated timepoints with 1 μM D/D solubiliser (FKBP AP21998) (Clontech). At the end of the timecourse the cells were trypsinised, fixed in 4% paraformaldehyde and resuspended in PBS-A. The cells were analyzed on a BD LSRFortessa analyser (BD Biosciences). Live, whole cells were identified using forward and side scatter parameters and the amount of the eGFP tagged reporter construct remaining at each time point was expressed as a percentage of the zero time point for each siRNA. FlowJo software was used for analysis. Western blot was used to determine level of protein knockdown.

EGF endocytosis and EGFR degradation assays

For EGFR degradation assay, HeLa cells were untransfected or transfected with siRNAs using protocol listed in secretion assay section and serum starved for 16 hours. The following day, individual wells were stimulated with 100 ng/ml EGF (Sigma-Aldrich) for the indicated times. After stimulation the cells were lysed in 150 ul/well TNTE (20 mM Tris pH 7.4, 150 mM NaCl, 5 mM EDTA, 1% Triton X-100 supplemented with protease inhibitor cocktail (Roche) and PhosStop (Roche)).

Lysosomal and mitochondrial function assays

For measurement of lysosomal pH, B6.*Clec16a*^{GT} MEFs were pretreated with positive controls and then incubated for 30 minutes with 4uM Lysosensor Yellow/Blue DND-160 (Life Technologies, Cat No. L-7545). Cells with rinsed with isotonic solution and then incubated with PBS or calibrated standards for 4 minutes (Coffey et al., 2014). Data was

collected on a Biotek Synergy H1 Hybrid Reader, measuring excitation at 340, 380nm and emission at 527nm. For enzyme activity assays, HeLa-*CLEC16A* cells were serum starved and supernatant was collected. Cell pellets were lysed in 1% TritonX-100 containing protease inhibitors (Roche, Penzburg, Germany), sonicated and centrifuged. Supernatants were used to measure both intracellular and extracellular enzyme activity levels for β-hexosaminidase, α-mannosidase and β-glucuronidase. For measurement of mtDNA, total DNA was extracted from B6.$Clec16a^{GT}$ MEFs. DNA was 10-fold serially diluted in tRNA and used as input for SYBR green qPCR analysis using mtDNA primers MT9 and MT11, described as in (Kolesar et al., 2013). Relative mtDNA quantities were confirmed to be similar between genotypes at 1ng, 10ng, and 100ng of DNA input using the $\Delta\Delta C_T$ method, with normalization to nuclear DNA using a Taqman qPCR assay for genomic Actin (IDT, Mm.PT.58.29001744.g).

Supplementary Figure Legends

Supplementary Figure 1. Characterization of $Clec16a^{GT}$ and $Clec16a^{CURT}$ mutant mouse strains.

(**a**) Schematic of the mouse Chr. 16 locus containing *Clec16a* and the genetic alterations associated with the $Clec16a^{GT}$ and $Clec16a^{CURT}$ mutant mouse strains. Gene symbols: *Ciita*= MHC class II transactivator, *Dexi*= dexamethasone-induced transcript, *Clec16a*= C-type lectin domain family 16, member A, *Socs1*= suppressor of cytokine signaling.

(**b**, **c** and **d**) Image demonstrating size dimorphism (**b**) and growth curves of male and female *Clec16a*^{GT} (**c**) and B6.*Clec16a*^{GT} (**d**) mice. Number of mice/group indicated in graphs.

(**e**) Quantification of RNA transcript copies (relative to 10^7 copies of *Actin*) of *Clec16a* and neighboring genes, *Ciita*, *Dexi*, and *Socs-1*, from B6.*Clec16a*^{GT} and *Clec16a*^{CURT} MEFs (n=3-5 experiments). The exon or exons for each gene targeted in qRT-PCR assays are indicated.

Data in **c**, **d**, and **e** represented as mean+/- s.e.m. Data were analyzed by one-way ANOVA (**c**, **d**) or unpaired Student's t-test (**e**), *P<0.5, **P<0.01, ***P<0.001, ****P<0.0001.

Supplementary Figure 2: Characterization of neurodegeneration in *Clec16a*^{CURT} mice and aged *Clec16a*^{GT} mice. (**a**, **b, c** and **d**) H& E staining from the pons/medulla, hippocampus, frontal lobe and basal ganglia of the brains of 8 week old *Clec16a*^{GT} mice (representative of n=4 mice/group).

(**e**, **f**) Calbindin and Bielschowsky staining of brain from representative 47 week old *Clec16a*^{GT} mice. Black arrows indicate "baskets" containing Purkinje cells in brain of WT/WT mouse or "empty baskets" lacking Purkinje cells in GT/GT mouse (**f**).

(**g, h, i** and **j**) H& E staining (**g**, **h** and **i**) and calbindin immunofluorescence (**j**) from brains of 8 week old *Clec16a*^{CURT} mice (representative of n=3 mice/group).

Supplementary Figure 3. Lack of Clec16a results in the presence of dystrophic axons and abnormal cellular structures in the cerebellum.

(**a**) Calbindin, p62 immunofluorescence staining from the deep cerebellar nuclei of 8 week old *Clec16a*^{CURT} mice (representative of n=3 mice/group).

(**b**) Transmission electron microscopy images of deep cerebellar nuclei tissue sections of 8 week old B6.*Clec16a*^{GT} mice. Images represent structures present in GT/GT mice only: dystrophic axons and abnormal cellular structures or membranes. (representative of n=3 mice/group). Autophagic structures indicated with black arrows.

Supplementary Figure 4: Generation and characterization of CLEC16A-mutant cells.

(**a**) Schematic of the human Chr. 16 locus containing *CLEC16A* and the genetic alterations associated with the CRISPR-generated CLEC16A-deficient HeLa cell clones. Gene symbols: *CIITA*= MHC class II transactivator, *DEXI*= dexamethasone-induced transcript, *CLEC16A*= C-type lectin domain family 16, member A, *SOCS1*= suppressor of cytokine signaling.

(**b**) Verification of loss of CLEC16A protein expression in indicated cells by western blot (representative of n=3 experiments).

(**c**) A representative western blot of p62 and LC3-II in indicated cells starved (starv) or treated with Bafilomycin A1 (BAF) (representative of n=3 experiments).

(**d** and **e**) Quantification of mtDNA by qPCR in B6.*Clec16a*^{GT} MEFs (**d**) and cellular ATP levels in B6.*Clec16a*^{GT} MEFs and HeLa-*CLEC16A* cells (**e**) (representative of n=3 experiments, data represent mean+/- s.e.m. and were analyzed by unpaired two-tailed Student's t-test, ns= not significant).

Supplementary Figure 5: Unaltered morphology of the endolysosomal system in CLEC16A-mutant cells.

(**a**) Confocal microscopy images of HeLa-*CLEC16A* cells stained for EEA1 (representative of n=3 experiments).

(**b**, **c**) EGF receptor degradation assay in HeLa-*CLEC16A* cells (representative of n=3 experiments).

Supplementary Figure 6: Mutation of *CLEC16A*, but not *Atg5*, results in altered morphology of the ER-Golgi intermediate complex and the Golgi apparatus.

(**a**, **b**, **c** and **d**) Confocal microscopy images of B6.*Clec16a*^{GT} (**a**), *Clec16a*^{CURT} (**b**) MEFs, HeLa-*CLEC16A* cells (**c**) and *Atg5* KO MEFs (**d**). Cells were stained for GM130, ERGIC-53, Giantin or TGN-46 (representative of n=3 experiments).

(**e**) A representative western blot of LAMP-1 in EndoH and PNGase digested B6.*Clec16a*^{GT} MEF lysates (representative of n=3 experiments).

Supplementary Figure 7: Accumulation of single membrane bound structures in CLEC16A-mutant cells.

(**a**) Transmission electron microscopy images of B6.*Clec16a*^{GT} MEFs (representative of n= 3 experiments). Red dotted lines indicate membrane bound structures containing cellular debris.

(**b** and **c**) Transmission electron microscopy (**b**) and cryo-immunoelectron microscopy (**c**) images of HeLa-*CLEC16A* cells immunogold labeled for LC3 and LAMP-1

(representative of n=3 experiments). Red dotted lines indicate membrane bound structures containing cellular debris.

Supplementary Figure 1

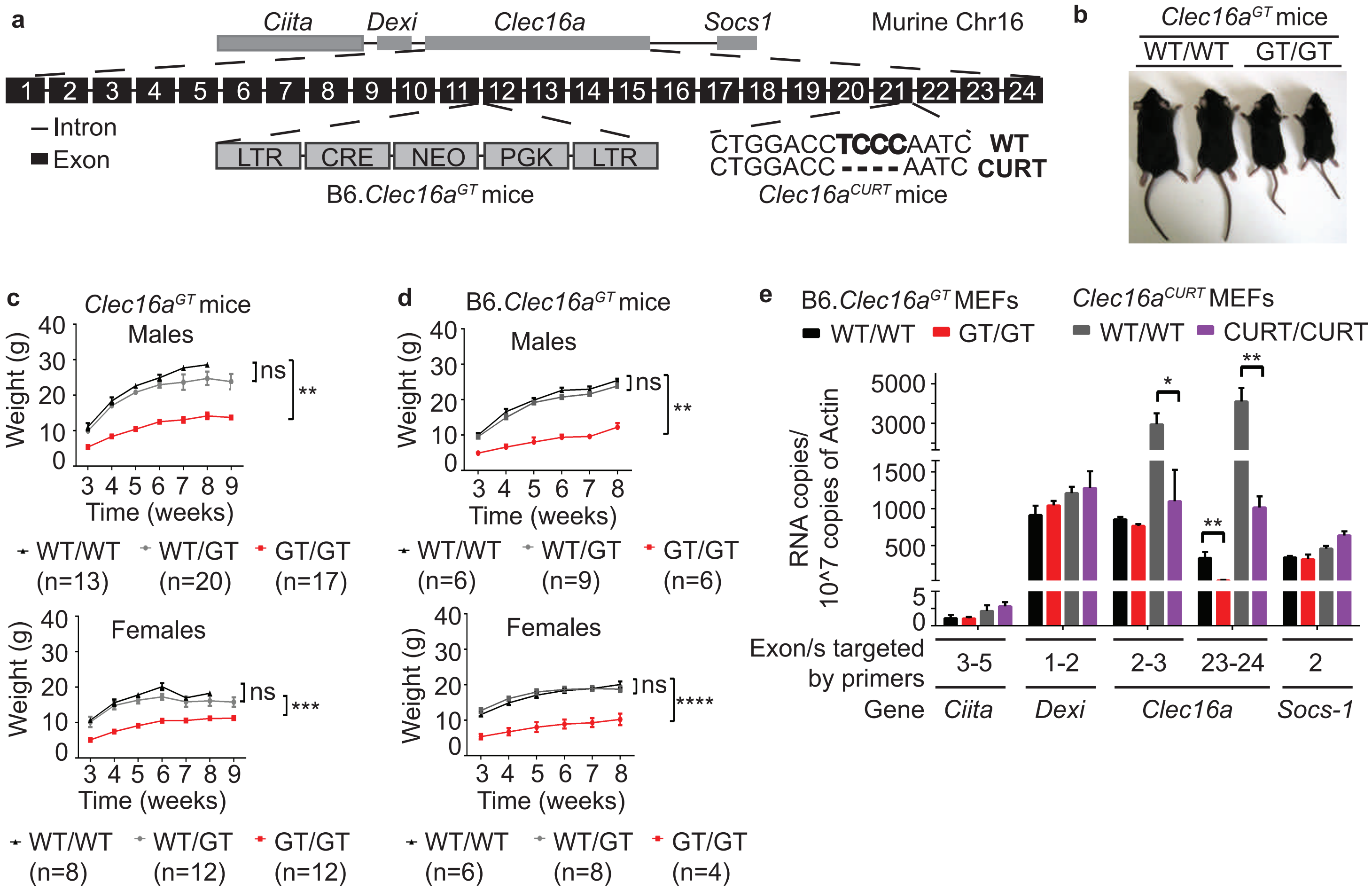

Supplementary Figure 2

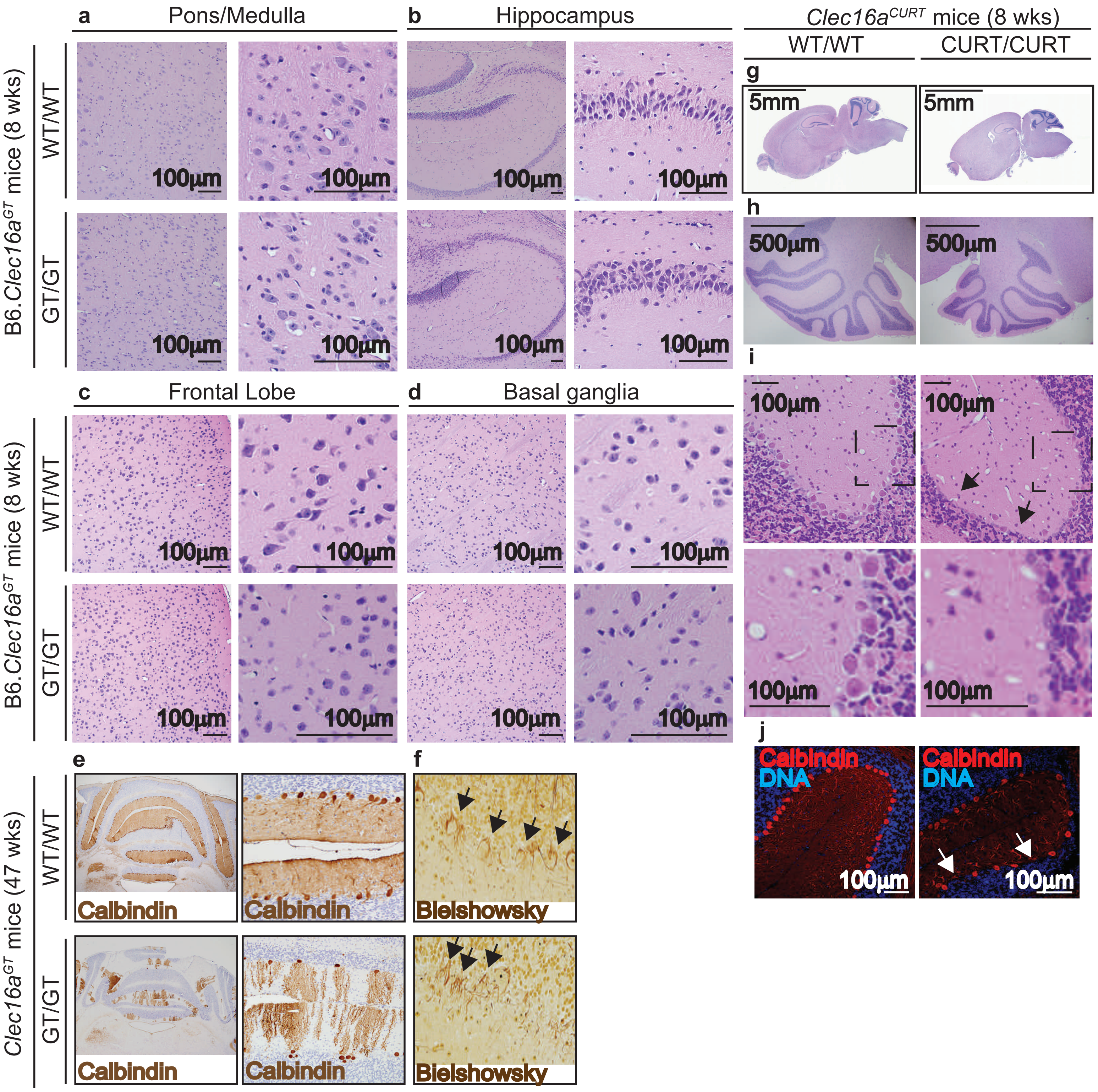

Supplementary Figure 3

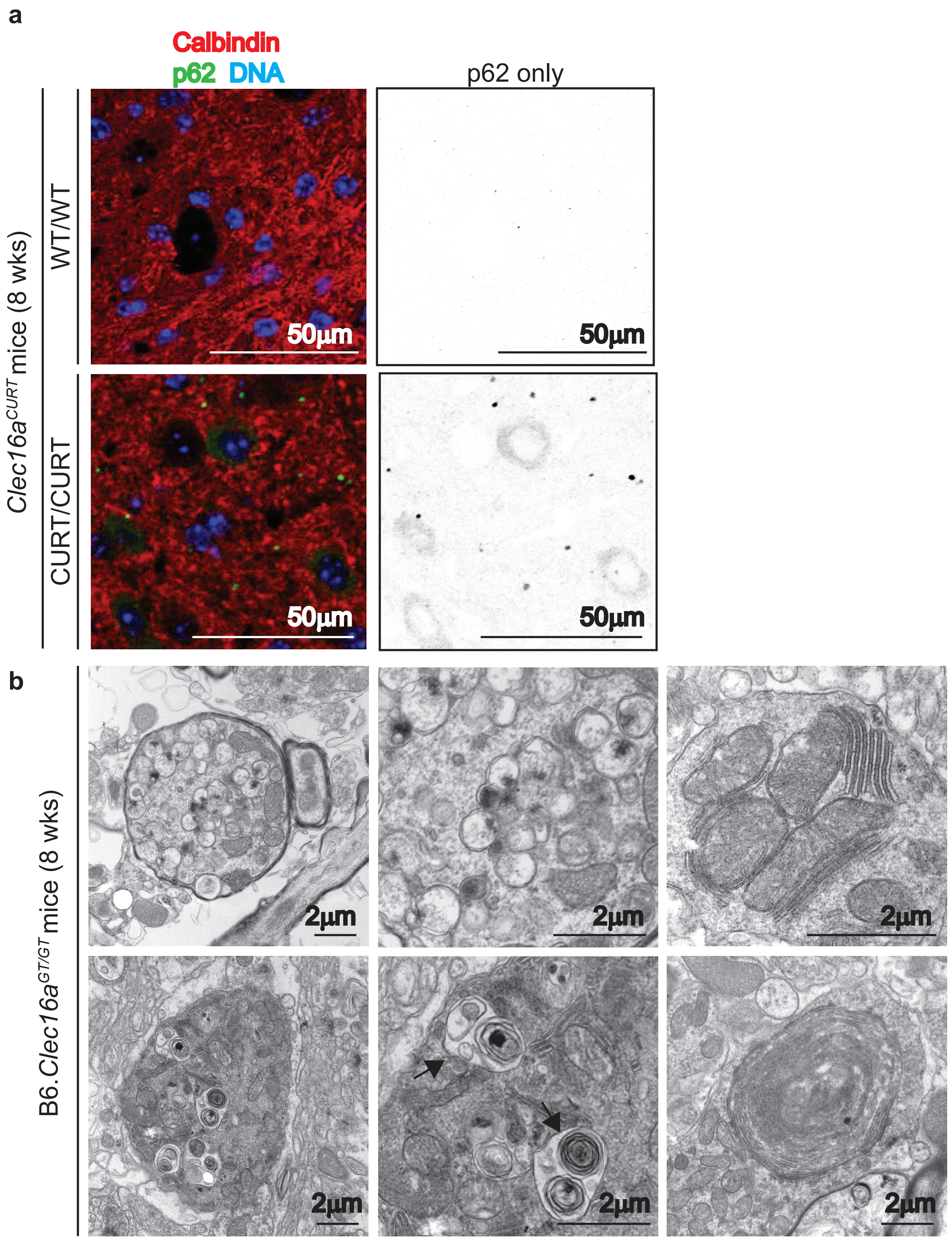

Supplementary Figure 4

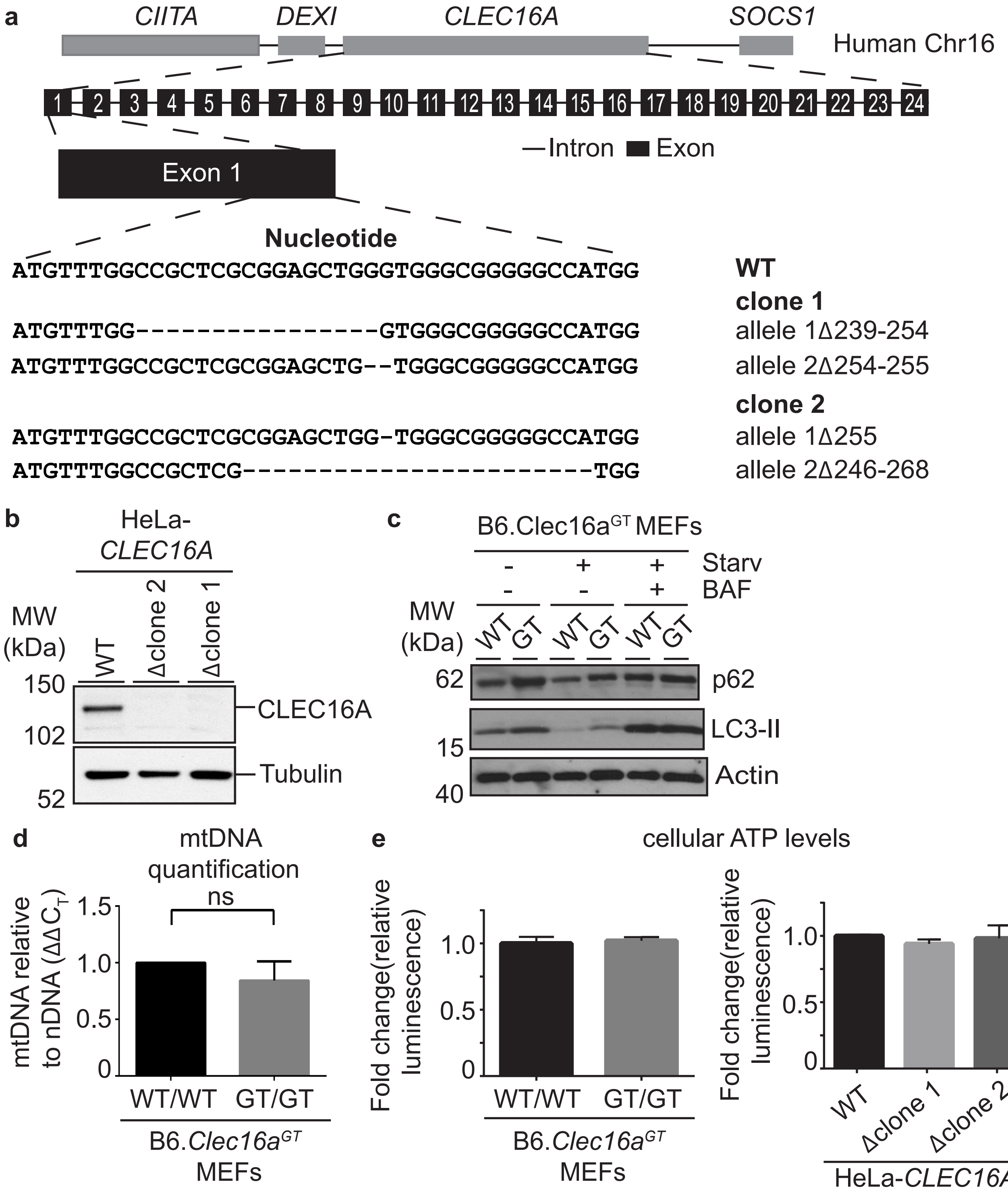

Supplementary Figure 5

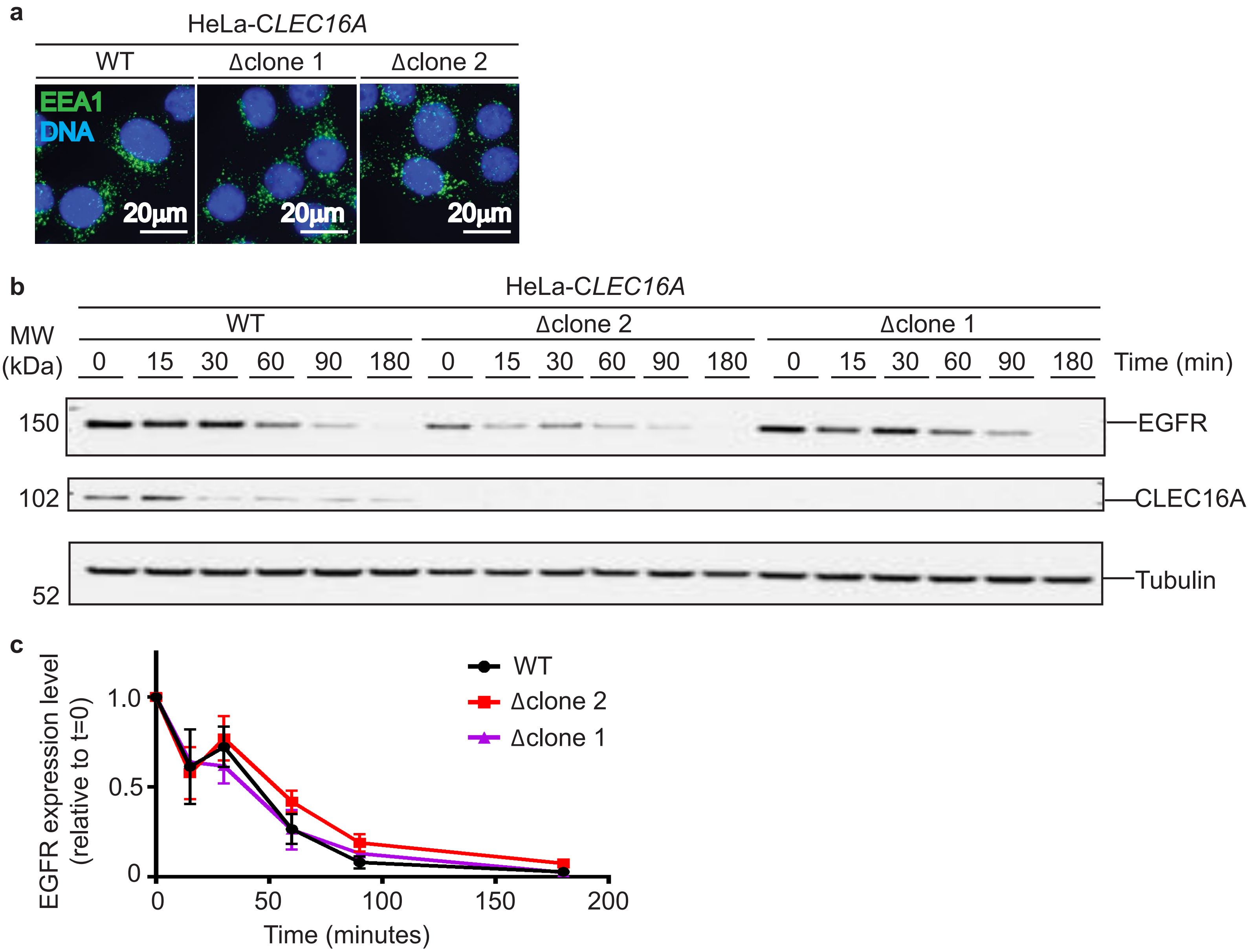

Supplementary Figure 6

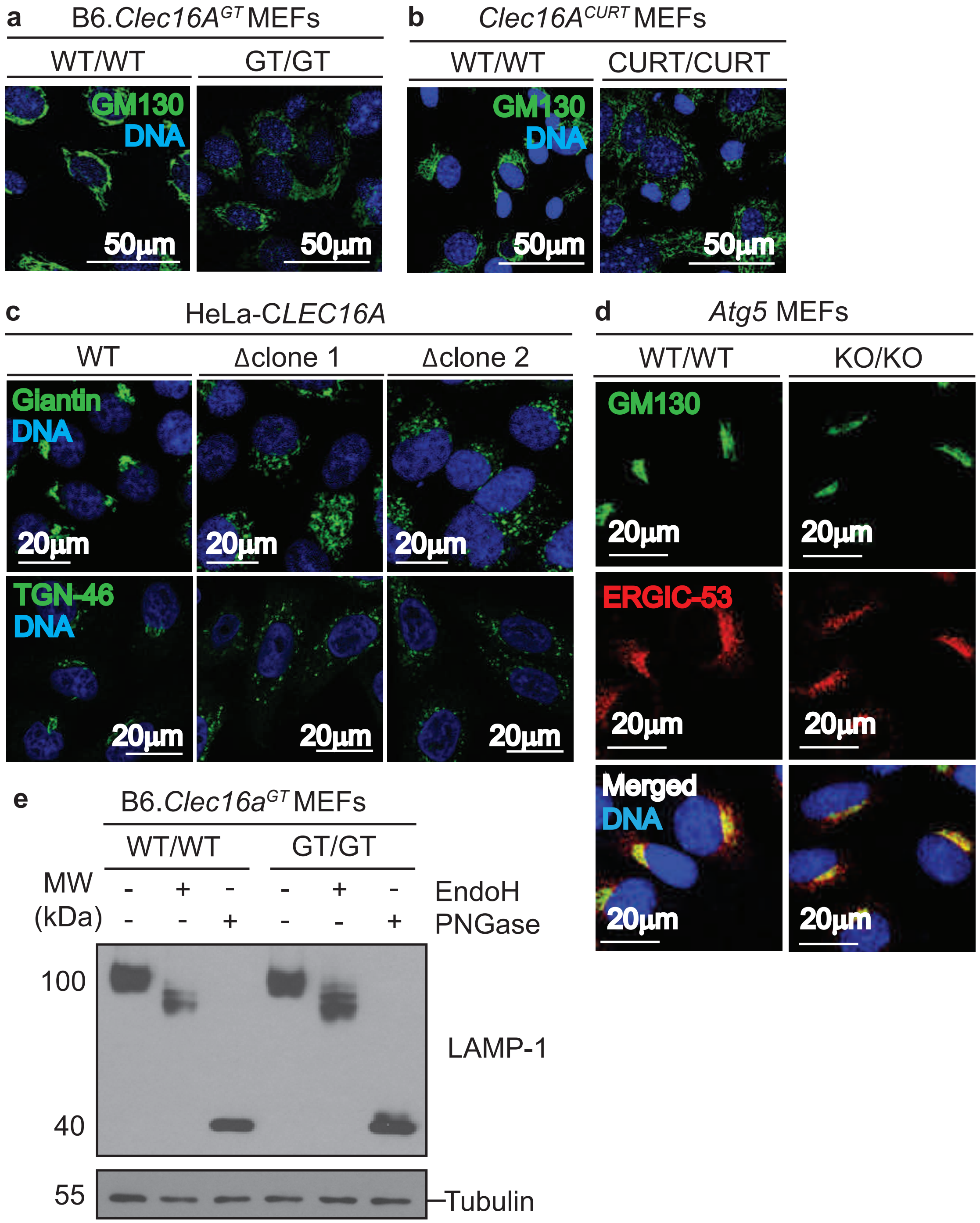

Supplementary Figure 7

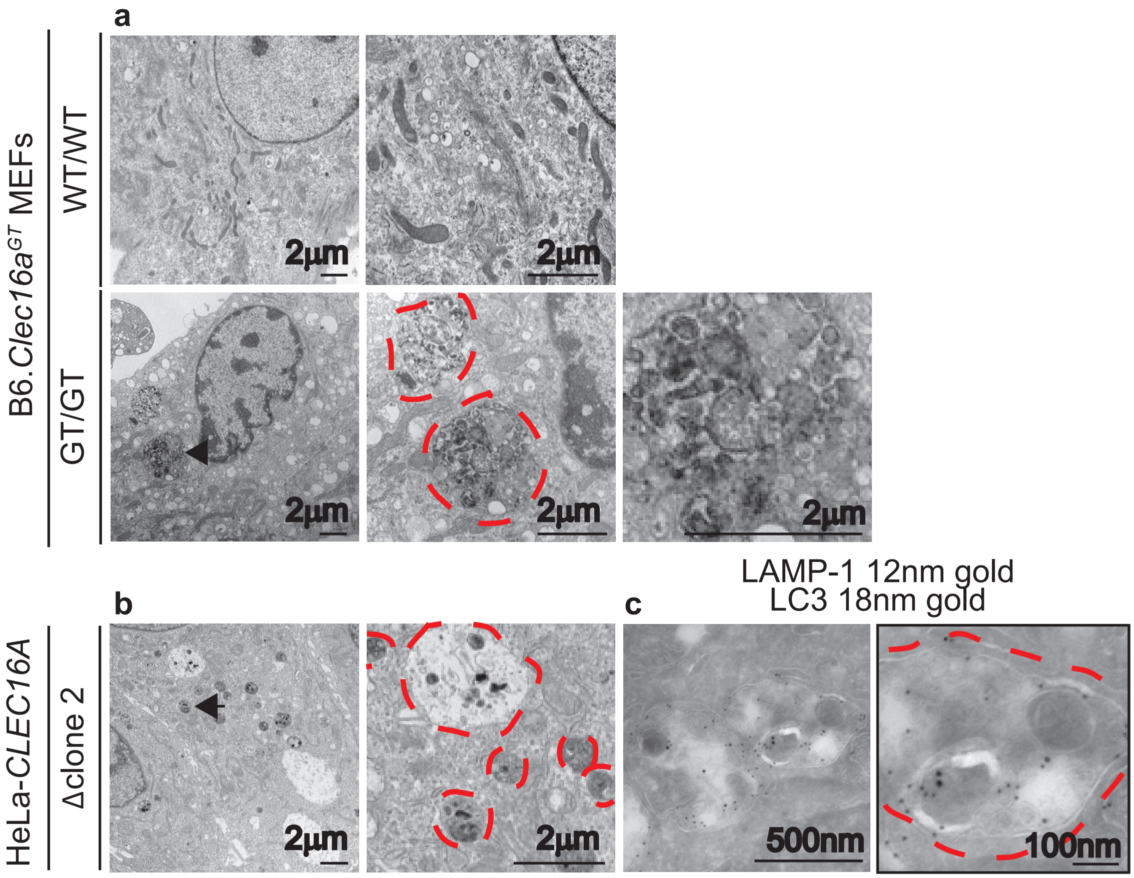

Supplement: Supplementary Information [file srep23326-s1.pdf]
